# Transcriptome analysis of *Colletotrichum nymphaeae*-Strawberry interaction reveals *in planta* expressed genes associated with virulence

**DOI:** 10.3389/fpls.2024.1390926

**Published:** 2025-01-20

**Authors:** Egem Ozbudak, Yisel Carrillo-Tarazona, Edinson A. Diaz, Flavia T. Zambon, Lorenzo Rossi, Natalia A. Peres, Sylvain Raffaele, Liliana M. Cano

**Affiliations:** ^1^ Indian River Research and Education Center, Department of Plant Pathology, Institute of Food and Agricultural Sciences (IFAS), University of Florida, Fort Pierce, FL, United States; ^2^ U.S. Department of Agriculture (USDA), Agricultural Research Service (ARS), U.S. Horticultural Research Laboratory, Fort Pierce, FL, United States; ^3^ Indian River Research and Education Center, Department of Horticultural Sciences, Institute of Food and Agricultural Sciences (IFAS), University of Florida, Fort Pierce, FL, United States; ^4^ Gulf Coast Research and Education Center, Department of Plant Pathology, Institute of Food and Agricultural Sciences (IFAS), University of Florida, Wimauma, FL, United States; ^5^ Laboratoire des Interactions Plantes Micro-organismes Environnement (LIPME), Centre National de la Recherche Scientifique (CNRS), Institut National de Recherche pour l’agriculture, l’alimentation et l’environnement (INRAE), Université de Toulouse, Castanet-Tolosan, France

**Keywords:** anthracnose, *Colletotrichum*, gene expression, strawberry, transcriptome

## Abstract

*Colletotrichum nymphaeae*, the causal agent of anthracnose fruit rot, is globally recognized as a major pathogen of strawberries due to its economic impact. Fungal pathogens utilize secreted proteins to facilitate infection by acquiring host nutrients and suppressing plant immunity. Understanding the transcriptomic responses of *C. nymphaeae* during infection can provide critical insights into its pathogenic mechanisms. In this study, RNA sequencing (RNA-seq) was performed to profile the transcriptome of *C. nymphaeae* strain 02-179 during infection of leaf and fruit tissues of the susceptible strawberry (*Fragaria x ananassa*) cultivar Florida Beauty. Differential gene expression analysis identified fungal genes upregulated during these interactions. Transcriptomic profiling revealed a set of genes encoding secreted effector proteins, including NUDIX hydrolase and LysM domain-containing proteins. Additionally, genes associated with Carbohydrate-Active enzymes (CAZymes), such as multicopper oxidase, pectinesterase, pectate lyase, glycosyl hydrolase family 7, and endochitinase, were significantly upregulated. Notably, two novel tannase genes were identified among the top upregulated genes in strawberry-infected leaves and fruits. Tannase enzymes are hypothesized to degrade tannins, a group of plant secondary metabolites abundant in strawberries, known for their defensive roles against pests and pathogens. The identification of tannase genes and the other genes associated with virulence underscores the complex molecular strategies employed by *C. nymphaeae* to infect and colonize strawberry tissues. Genes involved in degrading plant cell walls, suppressing host defenses, and potentially overcoming chemical barriers such as tannins play critical roles in the pathogenesis of anthracnose. Further functional characterization of these genes will enhance our understanding of the disease mechanisms and could inform the development of improved management strategies for *C. nymphaeae* infections in strawberries.

## Introduction

The genus *Colletotrichum* contributes to the major plant pathogens in the world that infect a wide range of species. Some *Colletotrichum* species are causal agents of anthracnose disease affecting various plant tissues and resulting in pre- and post-harvest crop damage. Plant disease caused by *Colletotrichum* species can result in enormous losses of crops worldwide by up to 100% (Baroncelli et al., 2017). The crops affected by *Colletotrichum* mainly include dicotyledonous plants such as strawberries, citrus, pome and stone fruits, and cereals like maize or sorghum. In addition to these crops, ornamentals, conifers or forage plants can also be a host to *Colletotrichum* species (De Silva et al., 2017; Sreenivasaprasad and Talhinhas, 2005).

While *Colletotrichum acutatum* sensu lato (s. l) in this genus can infect more than 90 genera of plants, which involve 100s of species, each cultivated plant family is susceptible to at least one *Colletotrichum* species (Baroncelli et al., 2017; De Silva et al., 2017; Sreenivasaprasad and Talhinhas, 2005). The lifestyle of fungi within the genus *Colletotrichum* is also variable and not limited to pathogenic. Although the most common lifestyle in the genus is hemibiotrophic, other lifestyles such as biotrophic, necrotrophic, and or endophytism have been described (Baroncelli et al., 2017; Gan et al., 2016).

*C. acutatum* s. l, the causal agent of anthracnose fruit rot, is recognized as the second most important pathogen of strawberries worldwide due to its economic impacts (Baroncelli et al., 2015). Plant species infected by *Colletotrichum* species generally show black acervuli on the dark-colored sunken lesions, hense anthracnose or “coal disease” designation (Ridzuan et al., 2018). The major pathogen causing anthracnose in strawberry plants worldwide is *Colletotrichum nymphaeae*, a member of the *Colletotrichum acutatum* (s.l.) species complex (Baroncelli et al., 2015; Dowling et al., 2020). The members of this species complex can infect different parts of strawberry plants, such as fruit, flowers, leaves, petioles, roots, or crowns. Fruit and flower parts are the most targeted and have the highest incidence of rot and blight symptoms, respectively, in the fields (Forcelini et al., 2018). Overall, these symptoms make the fruit unsaleable in the market and cause considerable yearly losses in the strawberry industry (Baraldi et al., 2015; Mertely and Legard, 2004). The initial preventive methods used to minimize the presence of this pathogen in strawberry include using disease-free transplants, selecting isolated localities to produce disease-free plants, improving air circulation, or using areas where environmental conditions are not favorable for disease (Staňková et al., 2011). However, as *C. acutatum* s.l. has become worldwide spread (Baroncelli et al., 2017; Mertely and Legard, 2004) and transplants that appear healthy may be the primary source of inoculum, and obtaining pathogen-free plants may not be practical anymore (Forcelini et al., 2018; Freeman, 2008; Mertely and Legard, 2004).

Standard control methods for anthracnose include the use of fungicides (Debode et al., 2015). In the past, some fungicides like dichlofluanid and a captan-benomyl mixture were reported to be effective against *C. acutatum* in New Zealand (Cheah and Soteros, 1984). However, factors like the enhanced demand for pesticide-free products (Debode et al., 2015), development of resistance in *Colletotrichum* spp (Forcelini et al., 2016), and environmental concerns, the continuous usage of some fungicides might not be sustainable, and they might no longer be reliable alternatives for disease control (Higuera et al., 2019).

Despite their economic importance, the genomic analysis of the *Colletotrichum* genus was overlooked, due to their complex taxonomy (Crouch et al., 2014). For *C. acutatum* s.l., it was not until 2016, that four common strains of the species complex (*C. nymphaeae, Colletotrichum simmondsii*, *Colletotrichum fioriniae* and *Colletotrichum salicis*) were sequenced (Baroncelli et al., 2016). The earlier Colletotrichum genomes that were sequenced have revealed the expansion of some gene families encoding Carbohydrate-Active enzymes (CAZymes), secondary metabolites, secreted proteases and small secreted proteins (SSPs) (Crouch et al., 2014). Particular CAZymes that degrade cells walls are called cell wall degrading enzymes (CWDEs), and they are especially used by hemibiotrophic or necrotrophic pathogens to penetrate their host cell and access the nutrients, with the combination of mechanical forces, reactive oxygen species (ROS) and toxins (Amil-Ruiz et al., 2011; Zeilinger et al., 2016). For that reason, they are an important part of the phytopathogenic arsenal and have been found to be highly expanded in *Colletotrichum* compared to other ascomycetes (Crouch et al., 2014; Gan et al., 2013; Tan et al., 2020).

Recent research by Ristinmaa et al. (2022) has identified several tannases that exhibit a preference for glucose-containing substrates, hinting at the possibility of tannases being included in the CAZyme database in the near future (Ristinmaa et al., 2022). Furthermore, feruloyl esterases, which belong to the same gene family as tannases, are already listed in the Carbohydrate Esterase Family 1 in CAZymes (Blackman et al., 2014). Tannase enzymes degrade tannins which are plant secondary metabolites that can be abundantly found in strawberries. During the pathogen attack, tannins can be induced as a phytoalexin and their protective roles in plants against pests or pathogens have been well known (Mamaní et al., 2012; Sharma, 2019). However, despite the antimicrobial or toxic effects of tannin, many fungi, yeast or bacteria can grow in tannin-rich environments owing to tannin degradation abilities of tannase. Tannase has been reported in major phytopathogenic bacteria (*Agrobacterium*, *Xanthomonas*, *Pseudomonas*, and *Erwinia*) (Sharma, 2019) and fungal species (*Aspergillus*, *Penicillium*, *Fusarium*, and *Sclerotinia* (Allan et al., 2019; Reges de Sena et al., 2014). In *Colletotrichum* spp., the tannase family was found to be highly expanded and associated with the detoxification of plant-based defense compounds (Liang et al., 2018). In a transcriptomic analysis of *Sclerotinia sclerotiorum*, two tannase-encoding genes, sscle_08g067140 and sscle_10g076570, were highly expressed during the infection in different host species, supposedly for the detoxification of plant-produced tannins (Allan et al., 2019). In addition to the detoxification roles of tannases, they can also cleave the cross-links in the polymers of the cell walls; therefore, they might act as CWDEs (Aguilar et al., 2007).

We performed RNAseq profiling of *Colletotrichum nymphaeae* 02-179 during infection in the leaf or fruit tissues of the susceptible strawberry (*Fragaria* x *ananassa*) cultivar Florida Beauty. To the best of our knowledge, this is the first RNAseq profiling of *Colletotrichum nymphaeae* during a strawberry infection. The data obtained from this research can provide important insights related to the disease mechanism of *Colletotrichum* pathogens for the identification of genes involved in pathogenicity.

## Materials and methods

### Plant material

Strawberry plants (cv. Florida Beauty), which are susceptible to anthracnose fruit rot disease, were provided by Dr. Vance Whitaker at University of Florida Institute of Food and Agricultural Sciences (UF/IFAS) the Gulf Coast Research and Education Center (GCREC) in Wimauma, Florida, USA. The plants were multiplied from stolons hydroponically under greenhouse conditions at the UF/IFAS Indian River Research and Education Center (IRREC) in Fort Pierce, Florida. When the plants reached the ideal size (approximately one month after planted from stolons), strawberry leaves were freshly harvested and rinsed with sterile water and used in the detached leaf infection assays. The detached fruit infection assay was performed at GCREC with strawberry fruit at 1/3 maturity stage freshly harvested from the field.

### Fungal material

*Colletotrichum nymphaeae* strain 02-179 was provided by Dr. Megan Dewdney from the UF/IFAS Citrus Research and Education Center (CREC) in Lake Alfred. *C. nymphaeae* strain 02-179 was collected from diseased strawberry fruit tissue in Dover, Florida, USA in 2002. Wang et al. (2019) identified strain 02-179 as *C. nymphaeae* within the species complex of *C. acutatum* (Wang et al., 2019).

### Fungal infection assays in strawberry

The leaves were detached and placed in square bioassay dishes (Thermo Scientific, catalog No. 12-565-224). We applied the detached assay rather than the attached assay to achieve control on the environmental conditions within the laboratory, which is supported by previous studies, such as those involving ‘Camarosa’ and ‘Strawberry Festival’ cultivars, by demonstrating a correlation between detached and assays and field trial outcomes (Forcelini et al., 2017). *Colletotrichum nymphaeae* strain 02-179 was cultured on potato dextrose agar (PDA) plates (Oxoid, catalog No CMO139) under optimal conditions (~25°C and 24 h light). After 7-10 days, fungal spores (conidia) were harvested, the spore concentration was counted via Haemocytometer, and the final concentration adjusted to 1x10^7^ spores/mL. For the wounded leaf assay, 20 μL aliquots of the spore solution were placed as droplets on leaves previously wounded with a 10 μL sterile pipette tip. In the unwounded leaf assay, leaves were inoculated with same concentration and volume of spores without prior wounding. Both sets of leaves were placed over wet autoclaved sterile papers in the bioassay dishes maintained under optimum infection conditions (~25°C and 14 h light) for symptom development. Three to four leaves were collected 1-, 2- and 3-days post inoculation (dpi) for the wounded leaf assay, and on 1-, 2-, 3-, and 5-dpi for the unwounded leaf assay. The 5 dpi samples from the wounded leaves were excluded due to early rotting because of wounding. However, in the unwounded leaves the samples remained fresh and for that reason 5 dpi samples were included. For each leaf, samples were taken with a No. 5 cork borer (11.2 mm diameter) from areas where spore droplets were placed. All leaf samples were immediately ground to a fine powder with liquid nitrogen using a mortar and pestle and stored at -80 °C prior to RNA extraction. The entire assay, including the mycelia control, was conducted with two biological replicates. Mycelia control was harvested from strain 02-179 grown on PDA plates after 7 days and treated in the same manner as the leaf samples. For the fruit infection assay, fresh strawberry fruit at 1/3^rd^ maturity were collected from the fields at GCREC in Wimauma, Florida. Harvested unripe fruit were sterilized by dipping into 0.3% household bleach solution for 3 minutes and rinsing with deionized water twice. For each time point sample, 12 fruit were placed into an egg carton, and cartons were placed in clean and transparent plastic boxes with lids. Each egg carton was placed in a separate box, and 10 cartoons/boxes were used. In total 120 strawberries (n = 10 x 12 = 120) were used for 4 time points and mock control of both biological replicates. To keep high humidity conditions in the boxes, 100 mL deionized water was added to the box. *C. nymphaeae* strain 02-179 was grown in PDA under optimum conditions (~25°C and 24 h light), and spores were harvested after 7 days. The final concentration of the spore solution was adjusted to 1x10^6^ spores/mL. An aliquot of 20 μL of the spore concentration was placed as droplets onto the strawberry fruit placed in the egg cartons and within plastic boxes. Then the boxes were incubated at optimum infection conditions (25°C and 14 h light) for the development of disease symptoms. Fruit samples were collected 1-, 2-, 3-, and 5-days post inoculation (dpi). For each collected fruit sample, the area where the droplets were placed was taken with a scalpel. Mycelia were also used as a control for fruit infection assays. Fruit tissues and mycelia samples were immediately ground to a fine powder with liquid nitrogen using mortar and pestle. Frozen powder samples were stored at -80°C prior to RNA extraction.

### RNA extraction, library preparation and sequencing

Total RNA extraction of strawberry infected tissues was performed with RNeasy^®^ Plant Mini Kit for each time point of the wounded leaf assay and the fruit assay according to the manufacturer’s protocol (Qiagen, Catalog No. 74903). To increase the RNA concentration, during the QIAshredder step, 4-6 tubes of the lysate samples were eluted into the same lilac collection tube. For the unwounded leaf assay, Spectrum™ Plant Total RNA Kit was applied for each time point according to the manufacturer’s protocol (Sigma-Aldrich, Catalog No. STRN50). RNA samples were digested with DNase enzyme following the manufacturer’s protocol to remove traces of DNA (Invitrogen TURBO-DNA-free kit, Catalog No. AM1907). We performed quality control on the total RNA samples by quantifying and measuring RNA purity (absorbance 260/280 ratios) with Nanodrop Lite and Qubit Fluorometer (v2.0). A total of 2 μg of total RNA for each plant tissue (wounded, unwounded and fruit infected samples) and time points were sent to Novogene Company (Sacramento, California) for cDNA library preparation (250-300 bp insert cDNA library size) and RNA-sequencing. Novogene used Illumina NextSeq500 platform for RNA-sequencing and generated paired-end (PE) read data of 150 bp in length. For each sample, we obtained around 6 Gb of read data. The raw read sequence data was deposited in SRA under accession PRJNA1074080.

### Secretome prediction and effector annotation

*Colletotrichum nymphaeae* SA-01 was used in this study as the reference species for secretome prediction and effector annotation. The *C. nymphaeae* SA-01 proteome sequences, and functional annotations were downloaded from the Joint Genome Institute (JGI) (https://genome.jgi.doe.gov/portal/Colny1/) and the Ensembl Fungi Repository (https://fungi.ensembl.org/Colletotrichum_nymphaeae_sa_01_gca_001563115/). Secreted proteins from *C. nymphaeae* SA-01 were identified by filtering the proteome for characteristics such as the presence of signal peptides, absence of transmembrane domains, and absence of Glycosylphosphatidylinositol (GPI) membrane anchor domains. We used SignalP v2.0 and v5.0 software to predict proteins carrying an N-terminal signal peptide leader sequence (http://cbs.dtu.dk/services/SignalP/) (Almagro Armenteros et al., 2019; Nielsen et al., 1997). We filtered out proteins with transmembrane domains using TMHMM v2.0 (https://services.healthtech.dtu.dk/service.php?TMHMM-2.0) and proteins with predicted GPI anchor domains using PredGPI (http://gpcr.biocomp.unibo.it/predgpi/pred.htm) and GPI-Som (http://gpi.unibe.ch/) (Pierleoni et al., 2008). We carry out the prediction of effector types (apoplastic or cytoplasmic) with EffectorP v2.0 (http://effectorp.csiro.au/) (Sperschneider et al., 2018). In addition, we run predictions for the subcellular localization of the secreted proteins with LOCALIZER (https://localizer.csiro.au/) which is based on the transit peptides (associated with chloroplast and mitochondria) and predictions of nuclear localization signals (NLS) (Sperschneider et al., 2017). CAZymes were predicted using CAZYme database on dbCAN2 server (Zhang et al., 2018a).

### RNAseq and gene expression analysis

**Figure 1:**
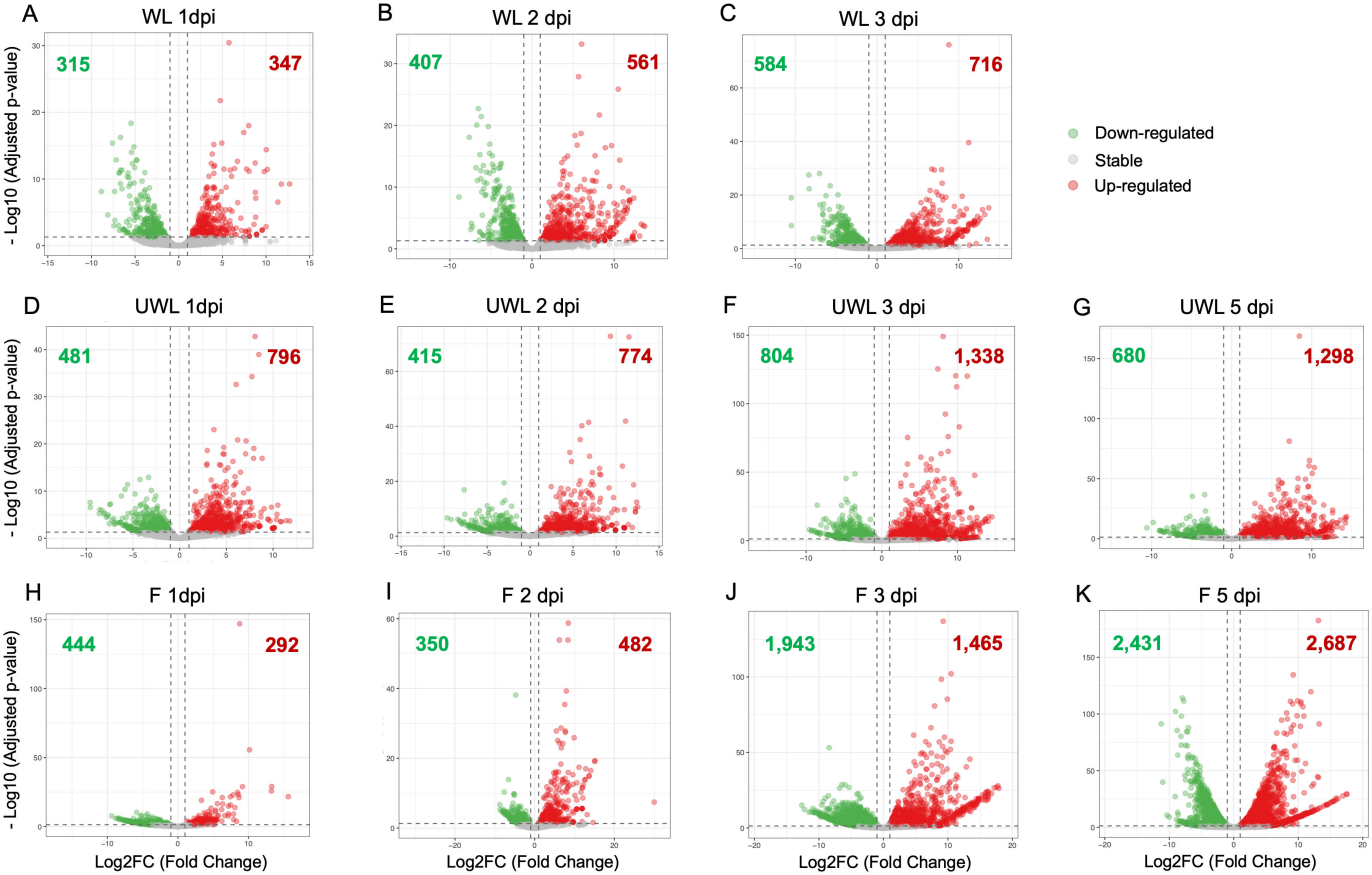
Volcano plots showing differentially expressed genes (DEGs) in *Colletotrichum nymphaeae* during infection of strawberry. Wounded leaf samples at **(A)** 1 dpi, **(B)** 2 dpi, and **(C)** 3 dpi. Unwounded leaf samples at **(D)** 1 dpi, **(E)** 2 dpi, **(F)** 3 dpi, and **(G)** 5 dpi. Fruit samples at **(H)** 1 dpi, **(I)** 2 dpi, **(J)** 3 dpi, and **(K)** 5 dpi.

**Figure 2:**
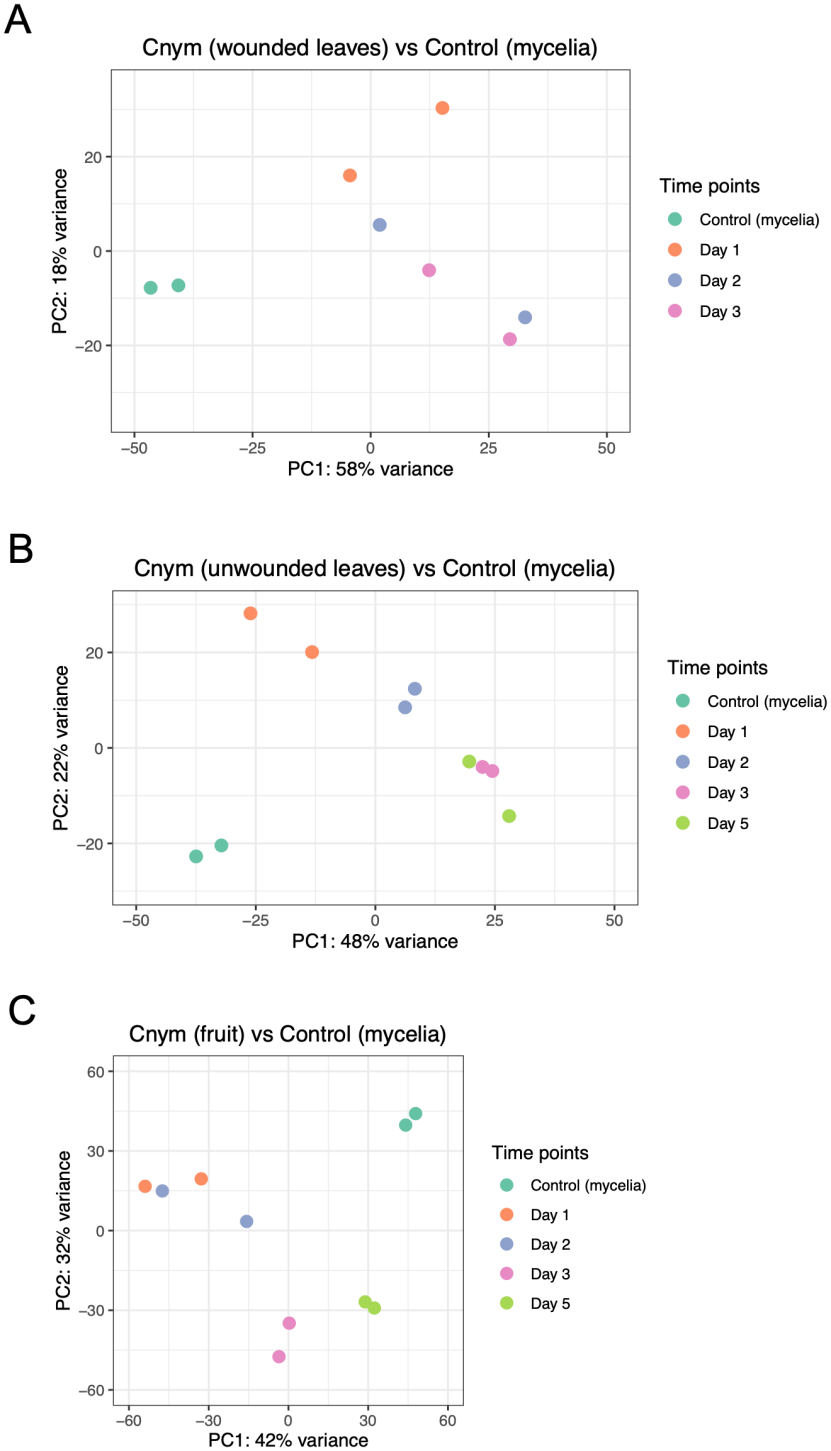
Principal component analysis plots showing correlation among replicates and treatments (strawberry infected tissues vs. fungal mycelia control samples of *Colletotrichum nymphaeae*). **(A)** Comparison of strawberry wounded leaf samples vs mycelia control. **(B)** Comparison of strawberry unwounded leaves vs. mycelia control. **(C)** Comparison of strawberry infected fruit samples vs. mycelia control.

**Figure 3:**
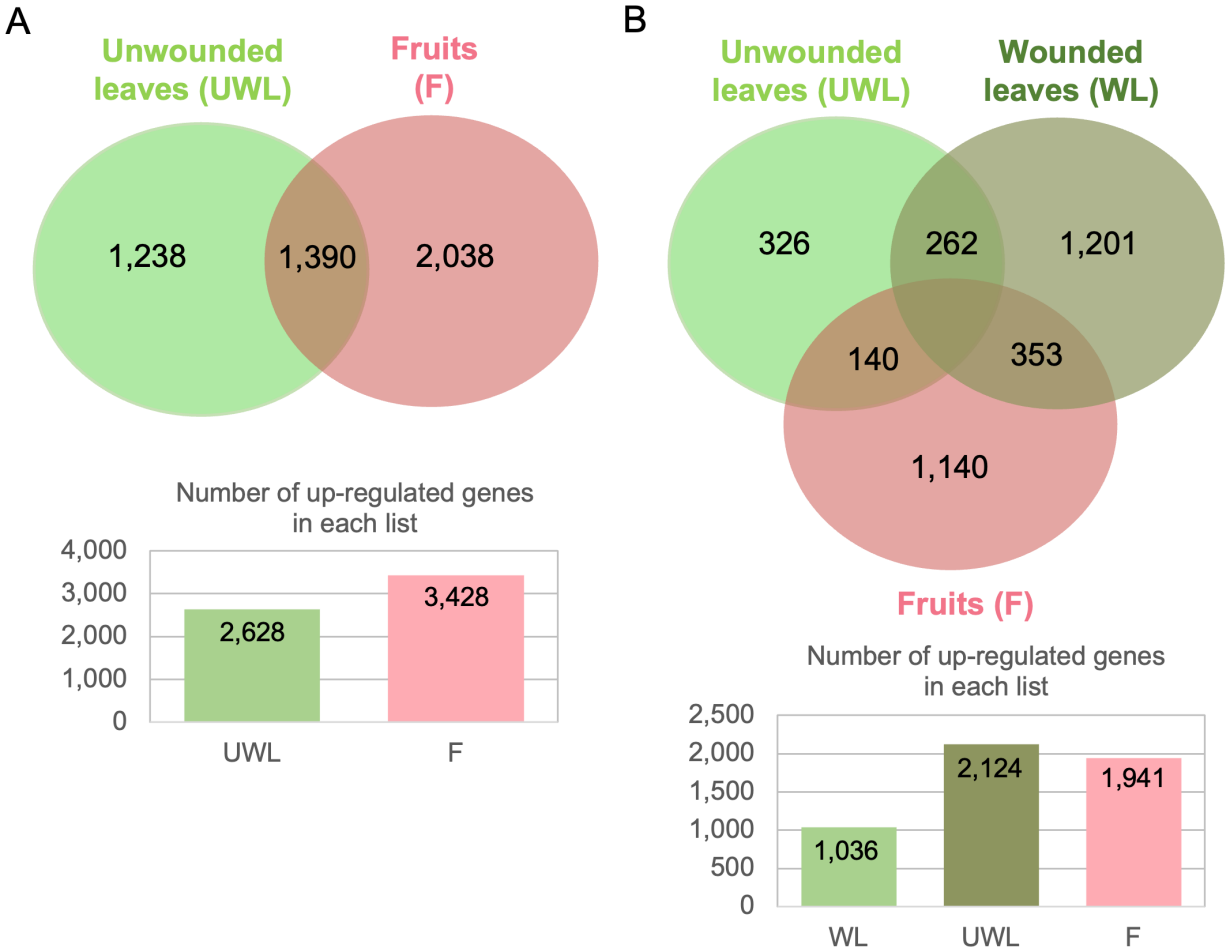
Venn diagrams illustrating the overlap of upregulated genes of *Colletotrichum nymphaeae* during infection on strawberry wounded leaf, unwounded leaf and fruit samples. **(A)** All time points are included. **(B)** 5 dpi time point is excluded.

Raw reads were processed with FASTX-Toolkit for quality check (Hannon, 2010). *Colletotrichum nymphaeae* SA-01 coding gene sequences (CDS) fasta files containing 14,404 sequences were used as the reference database for alignments. First, an index for *C. nymphaeae* SA-01 fasta CDS file was created with bowtie2-build function from Bowtie2 v2.2.6 software (Langmead and Salzberg, 2012). RNAseq reads from the three conditions (wounded and unwounded leaves, and fruit samples) were aligned to *C. nymphaeae* SA-01 using TopHat2 v2.1.1 software (Kim et al., 2013). The number and percentage of aligned reads for each condition are reported in **Table 1**. The output of the alignments in BAM (Binary Alignment Map) format was sorted by the read name with samtools-sort function (with –n parameter) from Samtools package (https://sourceforge.net/projects/samtools/files/) for downstream expression quantitation analysis. To count reads in features, we used htseq-count function (stranded parameter=no) from HTSeq v2.2.0 software (Putri et al., 2022). The raw counts from HTSeq were processed with DESeq2 package installed in RStudio to identify differentially expressed genes (DEG) (Love et al., 2014) by performing normalization using the median of gene count ratios relative to geometric mean per gene. Genes with Log2FC fold change (LFC) values greater than or equal to 1 and p-adjusted values less than 0.05 were considered significantly up-regulated. Genes with (LFC) values less than or equal to -1 and p-adjusted values less than 0.05 were considered significantly down-regulated (**Supplementary Table S1**). We searched for up-regulated genes among the differentially expressed gene list with known relevant functions associated with pathogenicity (**Table 2**). The expression of some selected genes based on the significant upregulation was further validated with reverse-transcription quantitative polymerase chain reaction RT-qPCR analysis (**Supplementary Figure S3**). The visualization of significant up-regulated and down-regulated genes was done with Volcano Plots using ggplot2 package in R (**Figure 1**) (Wilkinson, 2011). Variations between the time points and the control were checked with Principal Component Analysis (PCA) Plots using ggplot2 package in R (**Figure 2**). Comparisons of upregulated gene list among infected tissues were performed with E-venn (http://www.ehbio.com/test/venn/#/) and OrthoVenn2 tools (https://orthovenn2.bioinfotoolkits.net/home; **Figure 3**). Heatmap analyses were performed by publicly available Morpheus software (https://software.broadinstitute.org/morpheus) with a hierarchical tree analysis using one minus Pearson correlation method (**Supplementary Figure S1**).

**Table 1:** Summary of RNAseq read counts and mapping statistics of RNAseq read alignments of infected strawberry wounded leaf, unwounded leaf, and fruit tissues and control fungal mycelia samples to *Colletotrichum nymphaeae*.

Tissues	Sample IDs	No. of clean reads	Total mapped reads*	Mapped reads (%)
Infected strawberry wounded leaf (WL) samples with *Colletotrichum nymphaeae* 02-179 isolate	Cnym_WL_1dpi_A	22,963,751	592,707	2.5811
	Cnym_WL_1dpi_B	20,599,119	83,942	0.4075
	Cnym_WL_2dpi_A	23,174,411	519,584	2.2421
	Cnym_WL_2dpi_B	21,865,612	95,946	0.4388
	Cnym_WL_3dpi_A	20,140,124	565,844	2.8095
	Cnym_WL_3dpi_B	22,536,954	117,630	0.5219
Infected strawberry unwounded leaf (UWL) samples with *Colletotrichum nymphaeae* 02-179 isolate	Cnym_UWL_1dpi_A	19,804,463	17,826	0.0900
	Cnym_UWL_1dpi_B	22,417,896	27,010	0.1205
	Cnym_UWL_2dpi_A	19,837,603	23,692	0.1194
	Cnym_UWL_2dpi_B	20,015,406	24,765	0.1237
	Cnym_UWL_3dpi_A	23,676,199	53,460	0.2258
	Cnym_UWL_3dpi_B	26,283,584	53,908	0.2051
	Cnym_UWL_5dpi_A	25,592,499	32,661	0.1276
	Cnym_UWL_5dpi_B	22,357,028	30,858	0.1380
Infected strawberry fruits (F) samples with *Colletotrichum nymphaeae* 02-179 isolate	Cnym_F_1dpi_A	24,179,855	2,623	0.0108
	Cnym_F_1dpi_B	22,329,296	7,165	0.0321
	Cnym_F_2dpi_A	21,315,855	2,062	0.0097
	Cnym_F_2dpi_B	20,003,695	15,855	0.0793
	Cnym_F_3dpi_A	27,152,465	230,825	0.8501
	Cnym_F_3dpi_B	22,747,571	97,449	0.4284
	Cnym_F_5dpi_A	24,976,333	8,720,773	34.9161
	Cnym_F_5dpi_B	26,714,377	9,554,284	35.7646
Mycelia of *Colletotrichum nymphaeae* 02-179 isolate	Cnym_myc_A	29,653,534	13,193,400	44.4918%
	Cnym_myc_B	28,111,030	12,964,327	46.1183%

*Mapping reads statistics were obtained from the Tophat2 output files.

**Table 2:** List of *Colletotrichum nymphaeae* candidate upregulated genes in strawberry wounded leaf, unwounded leaf, and fruit tissues.

Gene ID	Description	Pfam/ Interpro domains^a^	Secreted^b^ Length (aa)	CAZyme^c^	Subcellular localization^d^	No. of Cysteines/%	Gene expression during infection in strawberries (Log2FC)^d^										
							Wounded leaves (WL) dpi			Unwounded leaves (UWL) dpi				Fruits (F) dpi			
							1	2	3	1	2	3	5	1	2	3	5
CNYM01_08295 (KXH55186)	Tannase	IPR011118/ PF07519	Yes, 588	–	–	10/2	8.83	8.61	7.13	–	–	10.61	9.13	13.24	12.82	8.72	5.04
CNYM01_13686 (KXH64601)	Tannase	IPR011118/ PF07519	Yes, 575	–	–	10/2	4.03	3.43	3.24	–	–	3.32	2.79	3.29	5.45	2.73	–
CNYM01_02731 KXH43592	Multicopper oxidase	IPR011707/ PF07732	Yes, 592	CAZy	–	9/2	–	7.49	8.42	–	–	8.54	8.32	8.56	8.67-	11.51	10.39
CNYM01_04268 (KXH39301)	Pectinesterase	IPR000070/ PF01095	Yes, 348	CAZy	–	4/1	–	10.76	11.04	–	–	11.66	12.03	–	–	10.84	7.68
CNYM01_03788 (KXH42765)	Pectate lyase	IPR004898/ PF03211	Yes, 254	CAZy	–	10/4	5.39	6.90	7.26	–	6.51	9.33	9.20	–	–	–	4.99
CNYM01_07125 (KXH45695)	NUDIX domain-containing protein	–	Yes, 380	–	–	3/1	–	–	4.41	–	–	6.96	6.22	7.14	6.51	8.48	4.45
CNYM01_07521 (KXH38331)	Glycosyl hydrolase family 7	PF00704/ IPR001223	Yes, 414	CAZy	–	5/1	2.30	3.48	4.01	–	4.41	5.17	5.89	4.96	6.20	6.55	3.99
CNYM01_09834 (KXH41657)	LysM domain	PF01476/ IPR018392	Yes, 160	–	Chloroplast, Y (0.881 | 22-62)	6/4	8.09	12.5	11.92	10.11	–	12.94	12.06	–	15.14	17.55	14.39
CNYM01_06500 (KXH30597)	Endochitinase	–	Yes, 750	CAZy	Nucleus, Y (PKPS)	18/2	5.18	3.12	–	5.78	3.7	–	–	–	–	–	–
CNYM01_11346 (KXH38857)	Cytochrome P450	IPR001128/ PF00067	No, 403	–	–	4/1	6.40	5.49	–	4.59	5.74	5.21	6.98	–	–	4.40	3.24
CNYM01_13494 (KXH42185)	Carboxylesterase	PF00135/ IPR002018	No, 526		–	5/1	–	–	9.11	–	–	10.77	–	–	11.96	14.15	9.52
CNYM01_02014 (KXH64700)	Chitinase-1	–	No, 477	–	–	0/0	–	–	–	–	–	–	–	9.15	8.08	5.5	3.54
CNYM01_07265 (KXH44404)	Major Facilitator Superfamily MFS transporter	PF07690/ IPR011701	No, 516	–	–	5/1	–	–	6.9	–	–	7.41	9.84	–	–	7.19	11.13
CNYM01_00135 (KXH60658)	Necrosis-inducing protein	PF05630/ IPR008701	No, 274		–	5/2	–	9.6	11.07	–	–	–	11.8	–	–	14.04	11.52
CNYM01_10757 (KXH57053)	NADPH2: quinone reductase	PF08240/ IPR013154	No, 383		–	3/1	–	10.98	11.18	10.85	–	9.85	11.69	–	–	15.29	12.49

^a^Pfam and Interpro domains: annotations predicted by the Joint Genome Instittue available at https://mycocosm.jgi.doe.gov/Colny1/Colny1.home.html.

^b^Secreted: predicton of secreted proteins using Signal P v2.0 and SignalP v5.0 webserver available at https://services.healthtech.dtu.dk/services/SignalP-5.0/.

^c^CAZyme annotations: for the prediction of carbohydrate active enzyme annotation CAZy we used dbCAN2 server available at http://cys.bios.niu.edu/dbCAN2.

^d^Subcellular localization annotations: fort the prediction of subcellular localizations of pathogen proteins we used Localizer program available at https://localizer.csiro.au/.

^d^Gene expression of *Colletotrichum nymphaeae* during infection in strawberries and at various days post infection (dpi). Log2FC, Log2 Fold Change values calculated by DESeq2.

### Validation of gene expression of candidate pathogenicity-related genes using RT-qPCR

The cDNA synthesis was performed using the SuperScript™ IV kit (Invitrogen, Catalog No.18091050). Total RNA samples were treated with TURBO DNA-free kit prior to cDNA synthesis (Invitrogen, Catalog No. AM1907). We first prepared a PCR tube per sample using: 1 µl 50 µM Oligo d(T), 1 µl 10 mM dNTP mix and 11 µl of template total RNA (250 ng). cDNA synthesis was carried out in a Thermal Cycler (Bio-Rad T100), the mixture was heated at 65°C for 5 minutes, and after that incubated on ice for at least 1 minute. In a second PCR tube, the reverse transcriptase reaction mix was prepared with 1 µl 100 nM DTT, 1 µl Ribonuclease inhibitor, 1 µl SuperScript™ IV Reverse Transcriptase (200 U/µl), and 4µl 5x SSIV Buffer. Then, the first and second tubes were combined, and the reaction mixture was incubated at 50°C for 10 min followed by inactivating the reaction by incubating at 80°C for 10 min. A quality check was performed on the single stranded cDNA samples. Quantification was performed with Nanodrop Lite and the absorbance A260/A280 ratios were measured with Qubit Fluorometer v2.0. RT-qPCR was performed on a StepOnePlus™ Real-time PCR system (Thermo Scientific, Catalog No. 4376600). For *C. nymphaeae* detection in this study, we designed specific primers for a total of eight *C. nymphaeae* candidate target genes: CNYM01_08295 (KXH55186) Tannase, CNYM01_13686 (KXH64601) Tannase, CNYM01_02731 (KXH43592) Multicopper oxidase, CNYM01_07521 (KXH38331) Glycosyl hydrolase family 7, CNYM01_06500 (KXH30597) Endochitinase, CNYM01_11346 (KXH38857) Cytochrome P450, CNYM01_03788 (KXH42765) Pectate Lyase, and CNYM01_07125 (KXH45695) NUDIX domain-containing protein. For *C. nymphaeae* housekeeping reference gene we designed specific primers for the Actin gene CNYM01_04906 (KXH42418) (**Supplementary Table S2**). The expression levels of the target genes of the fungal pathogen *C. nymphaeae* in the infection process were normalized to the constitutively expressed Actin reference gene CNYM01_04906 KXH42418) and were calibrated compared with the levels recorded in the control mycelia samples (set as 1) using the 2^–ΔΔCt^ method (Livak and Schmittgen, 2001) (**Supplementary Figure S3**). The quantitative qPCR assay was carried out in a final volume of 10 μL, consisting of 1 μL of cDNA template (from a 50 ng/μL stock to a final amount of 5 ng per 10 ul of SYBR green reaction), 0.5 μL of primer forward, and 0.5 μL of primer reverse (from 10 uM stock to a final concentration of 500 nM for each primer), 5 μL 2× Fast SYBR, and 3 μL of nuclease-free water. Three technical replicates were run for each sample.

### Gene ontology enrichment analysis

The gene ontology GO terms from *C. nymphaeae* strain SA-01 was downloaded from the Joint Genome Institute (JGI) (https://genome.jgi.doe.gov/portal/Colny1/). For the GO enrichment analysis, we used as input the list of up-regulated genes during infection and encoding for secreted proteins and load it into the SEA custom option from AgriGO Gene Ontology Analysis Toolkit http://systemsbiology.cpolar.cn/agriGOv2/c_SEA.php. We ran the GO enrichment analysis for three gene lists associated with the strawberry infected tissues (**Supplementary Table S3**). The GO enrichment statistical method used was Fisher test with a significance level threshold of 0.05.

### Phylogenetic analysis and active site prediction of Tannase family

Protein sequences of a set of 14 annotated tannase family members were retrieved from *C. nymphaeae* SA-01 proteome and combined in a multi FASTA format file. Additionally, we obtained protein sequences of two tannase genes, sscle_08g067140 and sscle_10g076570, from *Sclerotinia sclerotiorum* reported to be up-regulated during infection in different host species (*L. angustifolius* and *B. napus* respectively) from JGI https://mycocosm.jgi.doe.gov/Sclsc1/ (Allan et al., 2019). These two sequences were used as references then combined with the 14 tannase genes of *C. nymphaeae* SA01. The combined sequences underwent alignment using MUSCLE alignment function in MacVector 17.0.10 (Olson, 1994). Subsequently, a phylogenetic tree was constructed using the neighbor-joining method in MacVector. For the prediction of active sites in tannase enzymes, the I-TASSER server (https://zhanggroup.org/I-TASSER/) was employed, taking individual protein sequences as input (Zheng et al., 2021) (**Supplementary Figure S2**).

## Results

### RNAseq read alignments of infected strawberry samples with *Colletotrichum nymphaeae*

Transcriptomic analysis was performed from the RNAs extracted from the wounded and unwounded leaves, and fruit tissues of susceptible strawberry cultivar (*Fragaria* x *ananassa* cv. ‘Florida Beauty’) infected with *Colletotrichum nymphaeae* strain 02-179. RNAs were extracted from mycelia tissues grown *in vitro* conditions for the pathogen control and from the infected host tissues at 1, 2, 3, and 5 dpi. In wounded leaf samples, the percentage of paired reads mapped to *C*. *nymphaeae* SA-01 ranged between 1.35% to 1.65% on average. Although the alignment rate between time points only slightly changed, there was a significant difference between the biological replicates (**Table 1**). In the unwounded leaf samples, the alignment rate changed from 0.11% in the 1 dpi samples to 0.22% in the 3 dpi samples (**Table 1**). In the fruit samples, the paired read percentage dramatically increased from 0.02% in the 1 dpi samples to 35.35% in the 5 dpi samples. Moreover, unlike the wounded or unwounded leaf samples, a stable increase between each time point from the first time point (1dpi) to the last time point (5 dpi) was observed (**Table 1**). For mycelia controls, the alignment rate ranged between 44.49% to 46.12%. The paired reads were kept and applied for downstream analysis.

### Analysis of *Colletotrichum nymphaeae* genes encoding for secreted proteins and upregulated during infection in strawberry

Out of 14,404 proteins in the *C. nymphaeae* proteome, we identified 2,034 (14.12%) with secretion signals predicted with SignalPv2.0 and SignalPv5.0. After removing the proteins with predicted transmembrane domains (TM) and glycosylphosphatidylinositol (GPI) anchors, we identified a total of 1,517 (10.5%) secreted proteins (**Supplementary Table S1**). It is known that hemibiotrophic pathogens often possess a significant portion of secreted proteins in their proteomes, typically exceeding 10%. *Colletotrichum* spp. proteomes have been reported to exhibit a range of 13-16% secreted proteins (Crouch et al., 2014), which aligns with our findings. During the infection, we identified 268, 534 and 555 upregulated genes encoding for secreted proteins in the wounded leaves, unwounded leaves and fruits, respectively (**Supplementary Tables S1**, **S4**). Only 184 upregulated genes encoding for secreted proteins were commonly upregulated in wounded leaves, unwounded leaves and fruit samples (**Supplementary Tables S1**, **S4**). Out of these 1,517 secreted proteins, we found a total of 515 predicted as effectors with EffectorPv2.0. Out of the 515, 62 secreted effectors were commonly upregulated in wounded leaves, unwounded leaves and fruit samples (**Supplementary Tables S1**, **S4**).

### Differential gene expression analysis of *C. nymphaeae* during infection in strawberry tissues

For the wounded leaf samples, downregulated and upregulated gene numbers and percentage (in order) were 315 (2.19%) and 347 genes (2.41%) at 1 dpi, 407 (2.83%) and 561 genes (3.89%) at 2 dpi, and 584 (4.05%) and 716 genes (4.97%) at 3 dpi (**Figure 1**; **Supplementary Table S4**). In the unwounded leaf samples, downregulated and upregulated gene numbers and percentage (in order) were 481 (3.34%) and 796 genes (5.53%) at 1 dpi, 415 (2.88%) and 774 genes (5.37%) at 2 dpi, 804 (5.58%) and 1,338 genes (9.29%) at 3 dpi, and 680 (4.72%) and 1,298 (9.01%) genes at 5 dpi (**Figure 1**; **Supplementary Table S4**). For the fruit samples, downregulated and upregulated gene numbers and percentage (in order) were 444 (3.08%) and 292 genes (2.03%) at 1 dpi, 350 (2.43%) and 482 genes (3.35%) at 2 dpi, 1,943 (13.49%) and 1,465 genes (10.17%) at 3 dpi, and 2,431 (16.88%) and 2,687 genes (18.65%) at 5 dpi (**Figure 1**; **Supplementary Table S4**). The percentage of the upregulated and downregulated genes in wounded leaves gradually increased from 1 dpi to 3 dpi. However, in unwounded leaves, the percentage of both upregulated and downregulated genes did not show a steady increase. In the fruit samples, on the other hand, while the percentage of upregulated genes increased from 1 dpi to 5 dpi, the percentage of downregulated genes began to increase continuously only after 2 dpi.

After identifying upregulated genes from the samples at each time point, volcano plots were applied to visualize the data with the adjusted p-value and the Log2 Fold Change (LFC) value. Adjusted p-value were converted into –log_10_ value and plotted on the y-axis. On the x-axis, LFC values from the expression result are plotted and the threshold value was chosen as (-1) for downregulated genes or (+1) for upregulated genes. The greater expression values are represented closer to the left or right ends; the downregulated genes are shown in green color on the left side of the origin while the upregulated genes are shown in red color on the right side.

To check variation between the time points and the control, and assess quality, Principal Component Analysis (PCA) was applied using the read counts provided by HTSeq. In this analysis, closer distance between the points demonstrates more similar gene expression profiles of the samples (Sun et al., 2019). PCA results showed an accumulation of the points belonging to the samples from each time point of wounded leaves, unwounded leaves and fruits, away from the points belonging to the control group (**Figure 2**). This separation indicated the big differences between the control and sample groups in each assay. However, when it came to the variation within the time points in each assay, a different profile was observed. In the wounded leaves, despite their proximity, the same time points of the replicates were not necessarily grouped together which showed a variation between the biological replicates, which might be resulted from the wounding process. In the unwounded leaf and fruit samples, lower variation was observed which indicated the reproducibility of the replicates.

Considering all time points and all samples, we identified 4,877 upregulated DEGs, among which 514 (10.5%) core upregulated DEGs were common to fruits, wounded and unwounded leaves (**Figure 3A**). 68.01% of upregulated DEGs (3,317 genes) were specific to the infection of one plant organ with 1,449 upregulated genes during leaf infection only (wounded and unwounded), and 1,868 upregulated genes during fruit infection only, highlighting the prevalence of host colonization programs dedicated to each host organ. However, when the 5 dpi results (which were missing for the wounded leaf) were excluded from the diagram, significant changes were seen. When 5dpi time point was excluded, the number of upregulated DEGs belonging all remaining time points was 3,730 and the number of upregulated DEGs only in the wounded leaves, unwounded leaves, and fruits were 1,036 (27.77%), 2,124 (56.94%), and 1941 (52.04%), respectively. Therefore, compared to unwounded leaves and fruits, wounded leaves had lower number of upregulated genes (**Figure 3B**).

In the upregulated gene profile of all the samples, upregulation was observed for the genes that have previously been shown to be involved in pathogenicity in other *Colletotrichum* species (**Table 2**; **Supplementary Figure S1**). These genes included CAZymes involving enzymes with Auxiliary Activities (AAs) and different CWDEs such as pectinases, esterases and glycosyl (glucoside) hydrolases; SM related enzymes including cytochrome P450s, transporters and oxidoreductases; effectors associated with the switch to the necrotrophy such as NUDIX or NEP-1; and LysM proteins.

### Carbohydrate active enzymes

We identified 784 CAZyme genes in the *C. nymphaeae* genome through CAZyme prediction. The numbers of the upregulated CAZyme genes were as follows: 40 (5.10%), 65 (8.29%), and 83 (10.59%) on the 1, 2, and 3 dpi of the wounded leaves; 70 (8.93%), 102 (13.01%), 240 (30.61%), and 226 (28.83%) on the 1,2,3, and 5 dpi of the unwounded leaves: and 23 (2.93%), 41 (5.23%), 134 (17.09%) and 289 (36.86%) on 1, 2, 3, and 5 dpi of the fruits, respectively (**Supplementary Table S1**). The increase in the percentage of upregulated CAZymes from the beginning until the end of the infection in all three samples might be explained by their association with necrotrophy.

Multicopper oxidases are a subfamily of AA enzymes that are more recently integrated into the CAZyme database (Sützl et al., 2018). Two multicopper oxidase genes, KXH30257 and KXH43592, were highly upregulated in all the samples (**Supplementary Table S1**). KXH43592 exhibited a significant upregulation at the later stages for the wounded and unwounded leaves; respectively, by 7.4-fold at 2 dpi and 8.4-fold at 3 dpi, by 8.5-fold at 3 dpi and 8.3-fold at 5 dpi (**Table 2**). However, in the fruits it was upregulated at all time points; by 8.6-fold at 1 dpi, 8.7-fold at 2 dpi, 11.5-fold at 3 dpi and 10.3-fold at 5 dpi (**Table 2**; **Supplementary Figure S1**).

Another important group of CAZymes, CWDEs, were remarkably represented in the DEGs of all the samples. Carboxylesterase gene KXH42185 and glycosyl hydrolase family 7 gene KXH38331 were highly upregulated at the later stages in all samples. For KXH38331, LFC = 2.30 at 1dpi, LFC = 3.48 at 2 dpi, and LFC = 4.01 at 3 dpi in the wounded leaves, LFC = 4.41 at 2 dpi, LFC = 5.17 at 3 dpi and LFC = 5.89 at 5 dpi in the unwounded leaves, and LFC = 4.96 at 1 dpi, LFC = 6.20 at 2 dpi, LFC = 6.55 at 3 dpi and LFC = 3.99 at 5 dpi in the fruits (**Table 2**; **Supplementary Figure S1**). On the other hand, despite the similarities for the stage being expressed, leaf samples and fruits diverged with the different types of pectinases; pectin esterase gene KXH39301 was upregulated in the wounded (LFC = 10.8 at 2 dpi and LFC = 11.0 at 3 dpi) and unwounded leaves (LFC = 11.7 on 3 dpi and LFC = 12.0 at 5 dpi), meanwhile, in the fruits pectin lyase gene KXH42765 was upregulated on the late stage (LFC = 4.99 at 5 dpi; **Table 2** and **Supplementary Figure S1**).

**Figure 4:**
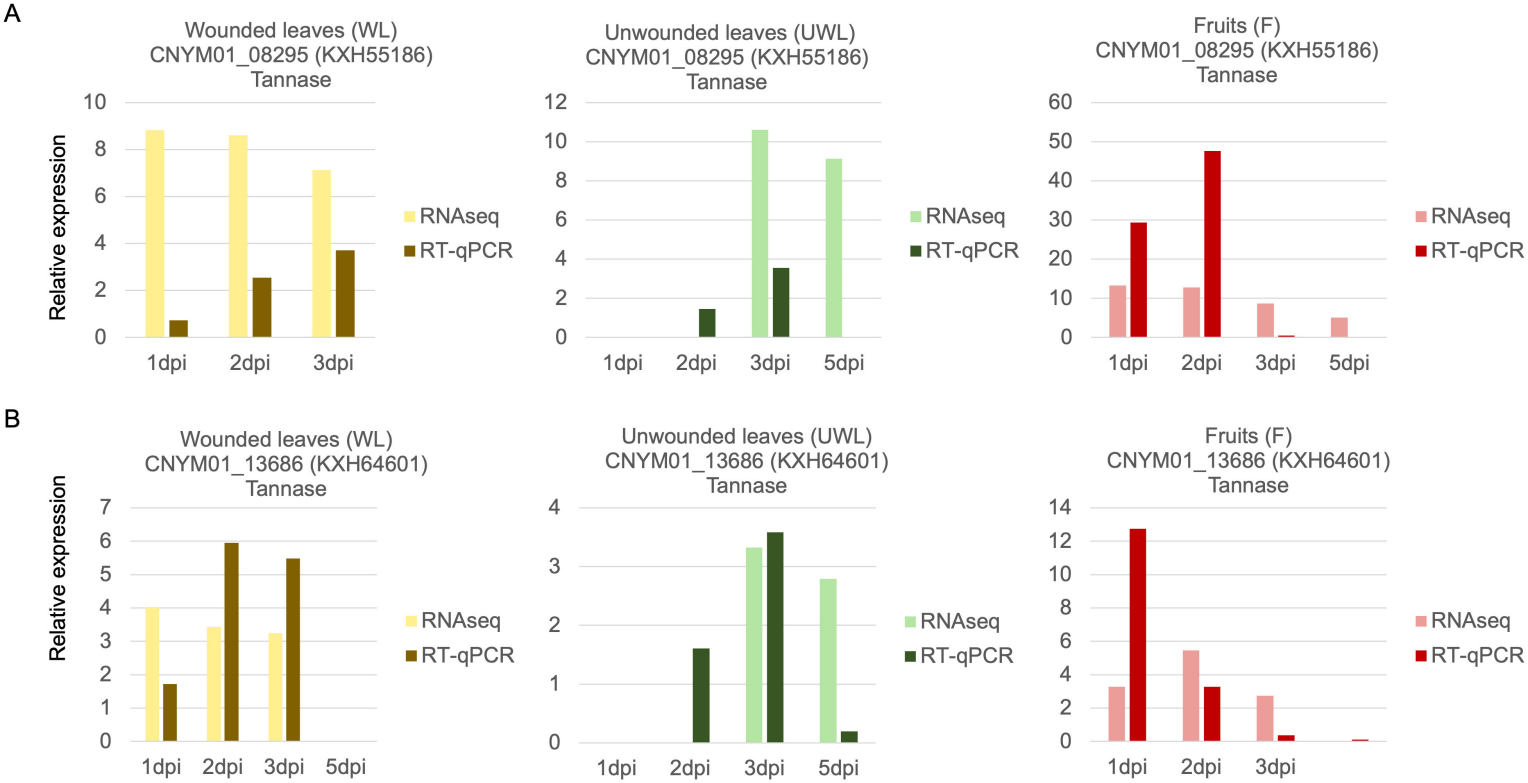
Comparison of RNAseq gene expression and reverse transcription-quantitative polymerase chain reaction (RT-qPCR) validation of two candidate genes of *Colletotrichum nymphaeae* encoding for Tannase proteins. **(A)** CNYM01_08295 (KXH55186). **(B)** CNYM01_13686 (KXH64601). The light green, light yellow and pink colored bars are used to illustrate RNASeq log2 fold change values in wounded leaves, unwounded leaves, and fruits, respectively. The dark green, dark brown and dark red colored bars are used to illustrate the RT-qPCR fold change mean values in wounded leaves, unwounded leaves, and fruits, respectively.

Among the up-regulated genes, one particular tannase gene, KXH55186, had very distinct and overlapping expression between all the samples and different time points: LFC = 8.8 at 1 dpi, LFC = 8.6 at 2 dpi and LFC = 7.1 at 3 dpi in the wounded leaves; LFC = 10.6 at 3 dpi and LFC = 9.1 at 5 dpi in the unwounded leaves; and LFC = 13.2 at 1 dpi, LFC = 12.8 at 2 dpi, LFC = 8.7 at 3 dpi and LFC = 5.0 at 5 dpi in the fruits (**Table 2**; **Supplementary Figure S1**). Alongside KXH55186, another tannase gene, KXH64601, was found to be significantly differentially expressed and had a different expression pattern compared to the other 12 tannase genes in the same tannase family (**Figure 4**).

### Secondary metabolism related genes

In differential expression analysis, upregulation of several secondary metabolite SM related genes, such as Cytochrome P450s, oxidoreductases and transporters, during the infection were found up-regulated at the later stages.

From the large family of Cytochrome P450 genes with 143 members, one of the most significantly upregulated ones in all the samples was KXH38857. Its expression was increased by 6.40-fold at 2dpi, 5.49-fold at 3 dpi in the wounded leaves, by 4.59-fold at 1 dpi, 5.74-fold at 2 dpi, 5.21-fold at 3 dpi and 6.98-fold at 5 dpi in the unwounded leaves, and by 4.40-fold at 3 dpi and 3.24-fold at 5 dpi in the fruits (**Table 2**; **Supplementary Figure S1**).

While ABC transporters show a significant upregulation only in the unwounded leaves (**Supplementary Table S1**), major facilitator superfamily (MFS) transporters were widely upregulated. MFS transporter gene KXH44404 was considerably upregulated: LFC= 6.9 at 3 dpi in the wounded leaves, LFC = 7.4 at 3 dpi and LFC = 9.8 in the unwounded leaves, and LFC = 7.2 at 3 dpi and LFC = 11.1 at 5 dpi in the fruits (**Table 2**). Among the upregulated SM related genes that overlapped between all the tissues were oxidoreductase genes. NADPH2:quinone reductase gene KXH57053 was one of the oxidoreductase genes that was immensely expressed in all the samples; LFC = 11.0 at 2 dpi and LFC = 11.1 at 3 dpi in the wounded leaves; LFC = 10.8 at 1 dpi, LFC = 9.9 at 3 dpi and LFC = 11.7 at 5 dpi in the unwounded leaves, and LFC = 15.3 at 3 dpi and LFC = 12.5 at 5 dpi, in the fruits (**Table 2**).

### LysM proteins

LysM proteins in DEG showed big differences at different stages of infection and diverged between the samples. Intracellular hyphae protein 1, encoded by KXH41657, showed very high upregulation in all the samples: LFC = 8.1 at 1 dpi, LFC = 12.5 at 2 dpi and LFC = 11.9 at 3 dpi in the wounded leaves; LFC = 10.1 at 1 dpi, LFC = 12.9 at 3 dpi and LFC = 12.1 at 5 dpi in the unwounded leaves; and LFC = 15.31 at 2 dpi, LFC = 17.6 at 3 dpi and LFC = 14.3 at 5 dpi, in the fruits (**Table 2**). Meanwhile, chitinase genes diverged between the samples; endochitinase gene KXH30597 was not differentially expressed in the fruits while chitinase-1 gene KXH64700 was only expressed in the fruit samples at all time points. LFC values of KXH30597 were 5.2 and 3.1 at 1 dpi and 2 dpi, respectively in the wounded leaves, and 5.8 and 3.7 at 1 dpi and 2 dpi, respectively, in the unwounded leaves. LFC values of KXH64700 were 9.1 at 1 dpi, 8.1 at 2 dpi, 5.5 at 3 dpi and 3.5 at 5 dpi in the fruits (**Table 2**).

### Necrotic-like proteins

Necrotrophic effectors in DEG like necrosis-inducing proteins and NUDIX domain-containing proteins were generally seen in the later stages of the infection. The most significantly expressed genes of these groups were, respectively, KXH60658 and KXH45695. In the wounded leaves, KXH60658 had increased expression at 2 dpi (LFC = 9.6 and at 3 dpi (LFC = 11.0); in the unwounded leaves the upregulation started at 5 dpi (LFC = 11.8), and in the fruits, it started at 3 dpi (LFC = 14.0) and continued at 5 dpi (LFC = 11.5; **Table 2**). KXH45695 had an increased expression at 3 dpi (LFC = 4.4) in the wounded leaves, and at 3 dpi (LFC = 7.0) and 5 dpi (LFC = 6.2) in the unwounded leaves. On the other hand, in the fruits general expressions including all time points were seen; LFC = 7.1 at 1 dpi, LFC = 6.5 at 2 dpi, LFC = 8.5 at 3 dpi and LFC = 4.4 at 5 dpi (**Table 2**).

### Phylogenetic analysis, prediction of ligand and active sites of *C. nymphaeae* tannase family

**Figure 5:**
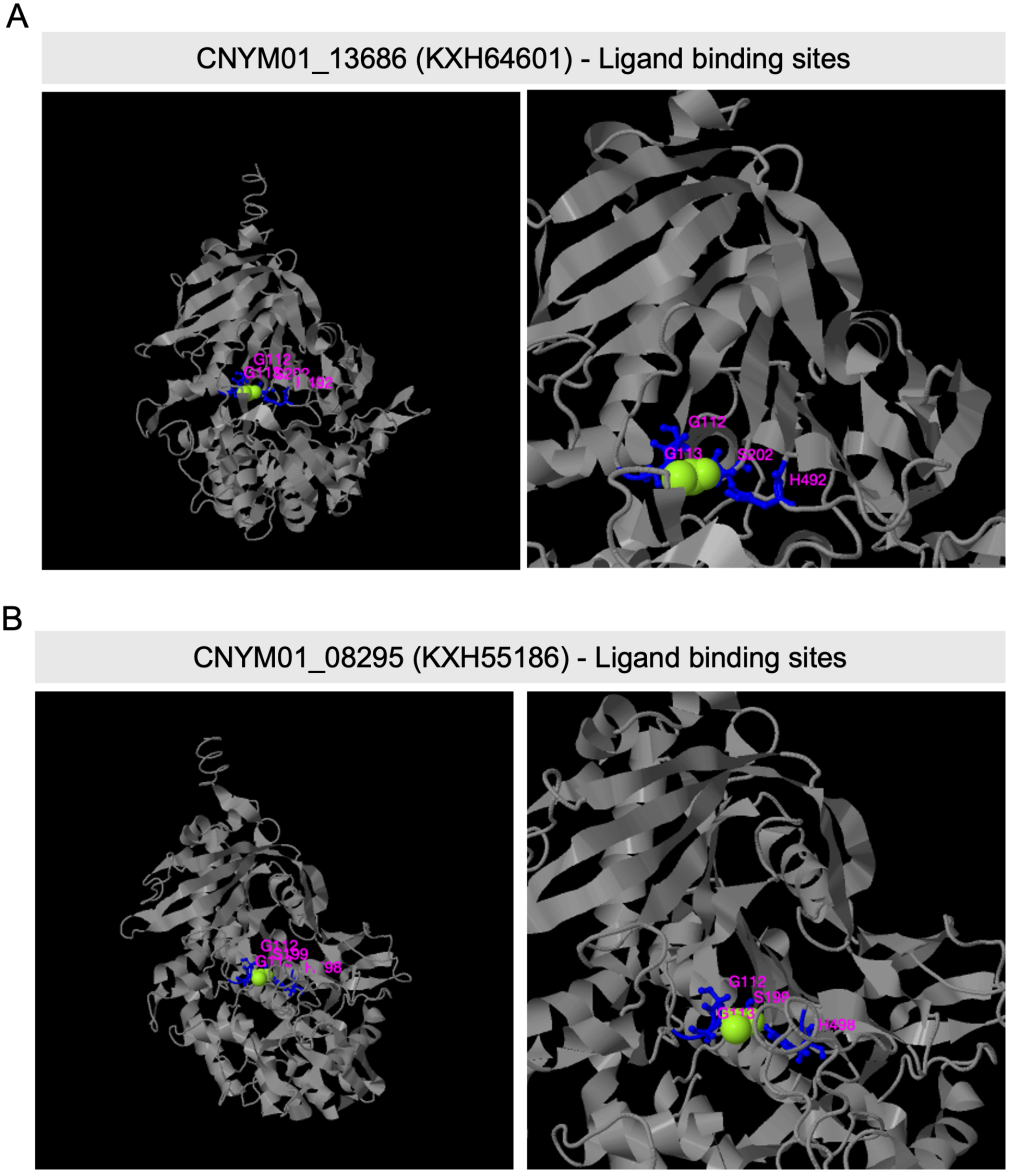
Predicted ligand binding site residues of two *Colletotrichum nymphaeae* Tannase proteins, by I-TASSER. **(A)** Predicted ligand binding site residues of CNYM01_13686 (KXH64601) with sites: G112, G113, S202, and H492). **(B)** Predicted ligand binding site residues of CNYM01_08295 (KXH55186) with sites: G112, G113, S199, and H498). The whole structure view is shown on the left and the zoom in view on the right. The ligand binding resides were predicted from PDB hit 4c01E comparison.

I-TASSER generated structure predictions for the protein sequences of two differentially expressed tannase genes (KXH64601 and KXH55186). These predictions were utilized to analyze the active site and ligand binding site residues of the enzymes. Serine and Histidine residues of the catalytic triad, Ser-Asp-His, associated with tannase enzymes identified in the ligand binding site of KXH64601 (**Figure 5A**) and KXH55186 (**Figure 5B**). In the case of the active site belonging to KXH64601, all three residues of the catalytic triad were predicted within the active site, but no prediction was obtained for the active site of KXH55186 (**Supplementary Figure S2**).

**Figure 6:**
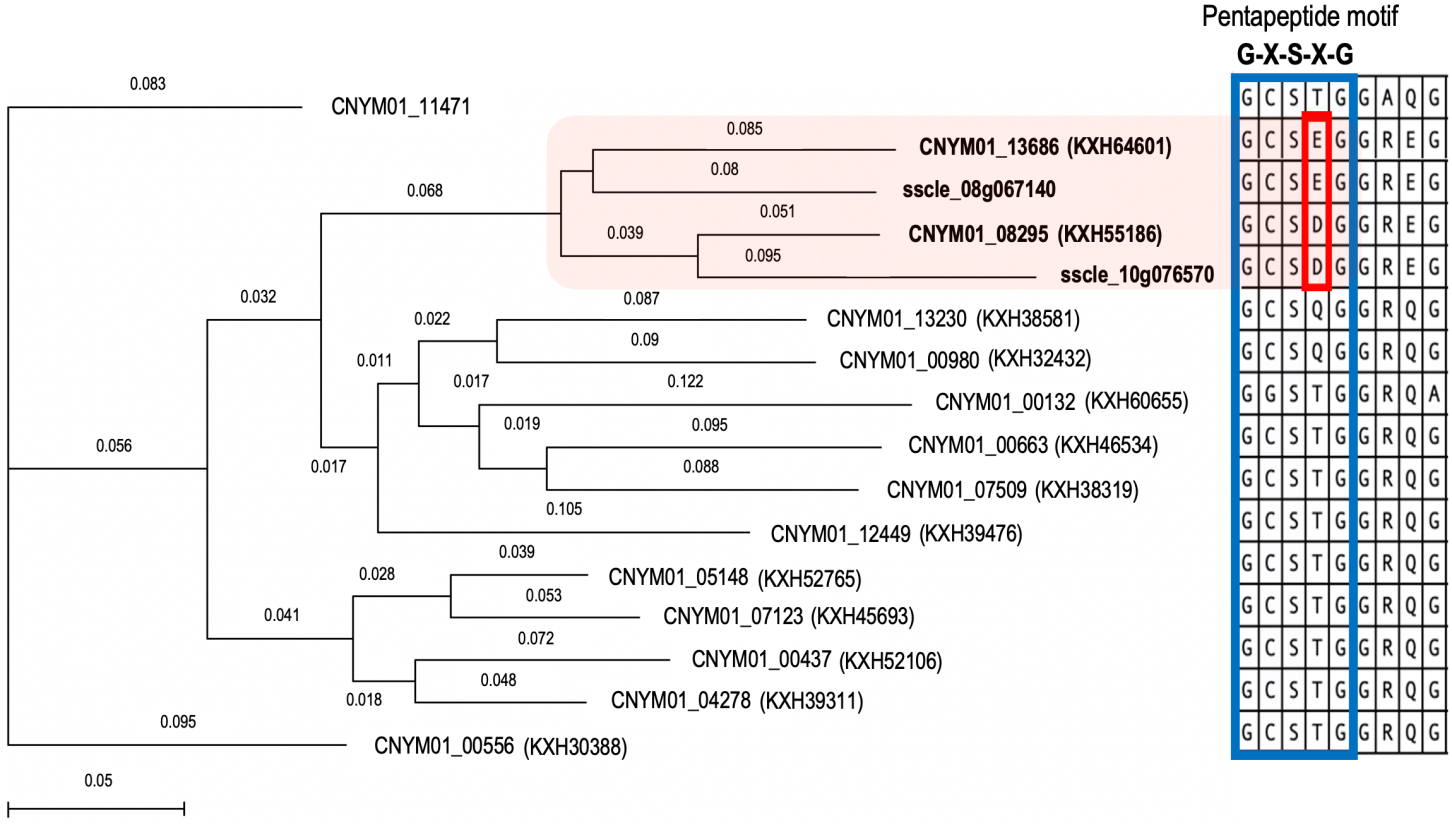
Phylogenetic analysis of the Tannase gene family members of *Colletotrichum nymphaeae* with reported *Sclerotinia sclerotiorum* tannases. Neighbor joining tree showing a conserved cluster of *Colletotrichum nymphaeae* Tannases and *Sclerotinia sclerotiorum* Tannases in the box in pink. Protein sequence alignment of a conserved region from *C. nymphaeae* and *S. sclerotiorum* Tannase family. The blue-lined box illustrates the pentapeptide motif which is conserved in all Tannases and the red-line box illustrates the specific aminoacid resides shared among *Colletotrichum nymphaeae* and *Sclerotinia sclerotiorum* tannases.

Multiple sequence alignment results showed the presence of the conserved pentapeptide motif “GXSXG”, where the serine residue of the catalytic triad is located, in all tannase sequences (**Figure 6**). However, the constructed phylogenetic tree, based on this alignment, formed a clade exclusively including tannase enzymes that were differentially expressed during infections. Moreover, the sequences in this clade had a different pattern in their pentapeptide motifs, having either “GCSDG” or “GCSEG” phenotypes while most of the remaining sequences had “GCSTG” phenotype.

### Validation of RNAseq gene expression using RT-qPCR

**Figure 7:**
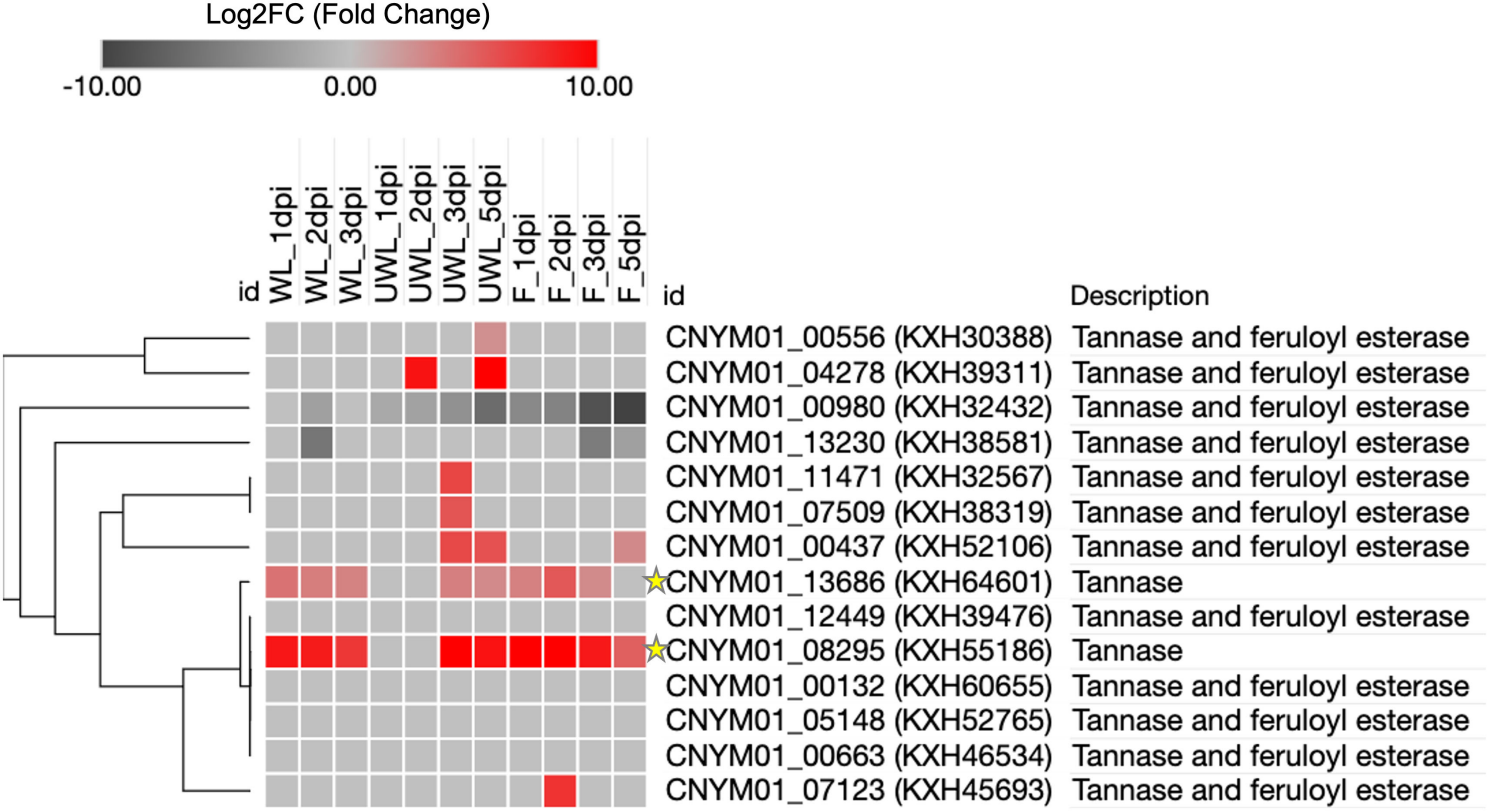
Heatmap of the Tannase gene family members of *Colletotrichum nymphaeae* during infection on strawberry wounded leaf, unwounded leaf and fruits samples. The colored bar illustrating the standardized Log2FC fold change from DESeq2. The green stars highlight the two Tannase genes which are up-regulated in all three conditions.

Validation of RNAseq gene expression results via RT-qPCR has been widely used in various transcriptomic analyses to confirm technical reproducibility. RNAseq analysis has a superior sensitivity than previous transcriptomic analysis techniques and does not necessarily apply probes which can cause bias (Wang et al., 2009). Nonetheless, RT-qPCR analysis can still be useful to validate RNAseq results as they can perfectly correlate. In our results, we were able to confirm the expression of two tannase genes (KXH64601 and KXH55186) (**Figure 7**), and other candidate virulence genes encoding for CAZYmes including multicopper oxidase (KXH43592), glycosyl hydrolase family 7 (KXH38331), endochitinase (KXH30597) and pectate lyase (KXH42765), an SM-related enzyme cytochrome P450 (KXH38857), in wounded and unwounded leaves, and fruits (**Supplementary Figure S3**).

### Gene ontology enrichment analysis

**Figure 8:**
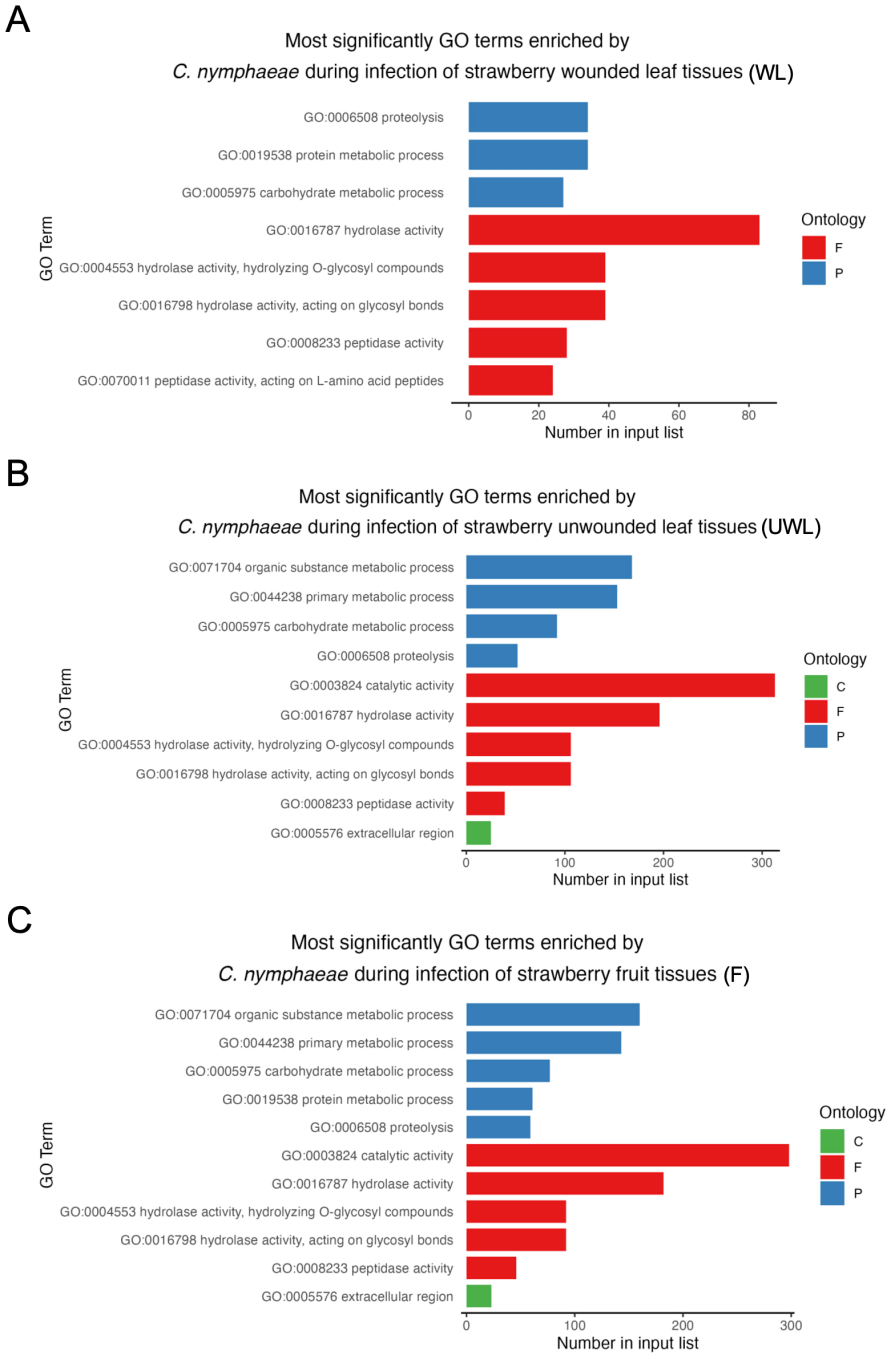
The most enriched GO terms of *Colletotrichum nymphaeae* during infection of strawberry. **(A)** Wounded leaves (WL). **(B)** Unwounded leaves (UWL). **(C)** Fruits (F). The three GO domains: biological processes (P), molecular function (F), and cellular component **(C)** are represented, respectively with blue, red, and green. The x-axis represents number of enriched GO terms and y-axis represents the GO term categories in three domains.

Gene ontology GO enrichment analysis was performed to determine the functional roles of in planta expressed genes in three groups: biological processes, molecular function, and cellular component (**Figure 8**). Total numbers of enriched GO terms in *C. nymphaeae* during infection in the wounded leaves, unwounded leaves and fruit were, respectively, 33, 62 and 61 (**Supplementary Table S3**). The most enriched GO term was in the molecular function domain in the wounded leaves, unwounded leaves and fruits. However, while the hydrolase activity was the most enriched in the wounded leaves, it was the second most enriched GO term in the unwounded leaves and fruits. The most enriched GO term was the catalytic activity for both unwounded leaves and fruits.

## Discussion

We performed RNAseq profiling of *Colletotrichum nymphaeae* 02-179 during infection in the leaf or fruit tissues of the susceptible strawberry (*Fragaria* x *ananassa*) cultivar Florida Beauty. The data obtained from this research can provide important insights related to the disease mechanism of *Colletotrichum* pathogens for the identification of genes involved in pathogenicity. To the best of our knowledge, this is the first RNAseq profiling of *Colletotrichum nymphaeae* during a strawberry infection.

As pathogen biomass is small relative to the host plant, especially during the early stages of infection, transcriptomic profiling of the pathogen can be inconvenient. Yet, Zhang et al. (2018b) conducted an RNAseq analysis of *Colletotrichum fructicola* in strawberry leaf infection and were able to recover important virulence genes from the mapped reads as small as 0.02 million reads (0.03% in percentage). In the first time points of results, we obtained much higher percentages of the mapped reads in the wounded and unwounded leaves (**Table 1**), while a similar ratio was observed in the fruits (**Table 1**). However, although the read map percentage for the leaf samples did not significantly change, fruit samples dramatically increased to 35.35% on the last time point. Human et al. (2020) found a correlation between fungal biomass and a switch to necrotrophy after conducting a time-coursed RNAseq analysis with a hemibiotrophic fungal pathogen, *Exserohilum turcicum* (Human et al., 2020).

In parallel with the increased fungal biomass in the fruit samples, the total and unique number of DEGs in the fruit were the highest in the samples (**Figure 3A**). However, when the 5 dpi results (which were missing in the wounded leaves) were excluded, the unwounded leaf samples had the highest number for the unique and total number of the genes (**Figure 3B**). This could be explained by the ripening process of the immature fruits at 1 dpi to become ripened towards 5 dpi. Unripe strawberries may not provide suitable conditions for fungi and the ripening process affects the availability of nutritional compounds for fungal growth. Therefore, the susceptibility levels in unripe strawberry fruits compared to the ripe are lower as the pathogens limit their growth and can remain in quiescent mode until being exposed to ripe fruit (Guidarelli et al., 2011; Prusky and Lichter, 2007). Moreover, the cell walls of ripened strawberries have been reported to be more susceptible to CWDEs of *Colletotrichum acutatum* (Amil-Ruiz et al., 2011). Considering this information, the significant increase in the number of DEGs can be explained by the increased susceptibility after the ripening of fruit samples. On the other hand, the number of DEGs in the wounded leaf samples remained much lower even after excluding the 5 dpi results. Liang et al. (2018) reported that while *Colletotrichum acutatum* s.l. pathogens can persist in quiescent mode, wounding of the host tissue might trigger colonization (Liang et al., 2018). Accordingly, it can be concluded that the wounding practice may eliminate a considerable number of DEGs as the pathogen can readily start colonization without any establishment.

Previous transcriptomic profiling of *Colletotrichum* species have shown distinguished expression of certain gene families like SSPs, CAZymes, SM related enzymes, and necrotrophic effectors during the infection in many different host systems. Our results with the expression profiles of these genes/gene families were parallel with the previous reports (Crouch et al., 2014; Dubrulle et al., 2020; Kleemann et al., 2012; O’Connell et al., 2012; Shang et al., 2020; Takahara et al., 2016; Zhang et al., 2018b).

The initial stages of the infection are characterized by the deployment of SSPs or biotrophic effectors to establish virulence in the host tissues by manipulating or suppressing the plant immune system. Therefore, pathogens at the biotrophic stage can maintain a prolonged relationship with their hosts while accessing the nutrients with minimum damage to the host cells (Kim et al., 2016). For instance, LysM effectors have various functions in the establishment and maintenance of biotrophy, such as dampening the host defense molecules or protection from plant chitinases through chitin sequestering (Tsushima et al., 2021). In parallel with this, proteins with LysM domains like intracellular hyphae protein-1 (KXH41657), endochitinase (KXH30597), and chitinase-1 (KXH64700) were significantly expressed starting from the early stages of the infection (**Table 2**). In *C. higginsianum*, LysM proteins were found to have further dual roles in appresoria formation in addition to the chitin sequestering or suppressing PTI responses. For that reason, basal expression of LysM proteins in the other stages besides biotrophy was associated with dual roles (Takahara et al., 2016). Likewise, in our results, expression of some LysM encoding genes was limited to the early stages while other genes like KXH41657 had more general expression., which could be explained by the presence of the dual roles.

CAZymes, SM related enzymes and necrotrophic effectors are considered as trademarks of necrotrophy as their gene families are more expanded in necrotrophs and a large array of them was secreted during the necrotrophic stages (Allan et al., 2019). CAZymes are employed by the fungal pathogens mainly for degrading the plant cell walls and the usage of their components as nutrients but also for degradation/modification of fungal cell walls, storage and protein glycosylation (Dubrulle et al., 2020; Kim et al., 2016). However, as in the initiation of the infection, pathogens need to penetrate the cell walls of the host and modify their own cell walls to evade PTI (which could be activated with chitins), particular CWDEs were observed in the initial stages of some *Colletotrichum* spp. As pectin is a major component of the plant cell walls and degradation of it would make the plant cell wall more accessible, these CWDEs comprise pectinase enzymes such as carbohydrate esterase (CE Family 1), glycosyl hydrolase (GH Family 3, 28 and 31) and pectate lyases. These enzymes can also pave the way for other CWDEs by degrading pectin in the plant cell walls (Zhang et al., 2018b). GH superfamily includes strikingly divergent members and in addition to pectin, they have functions in the hydrolysis of different cell wall components including cellulose, hemicellulose and beta-glycans (Allan et al., 2019). One of the most significantly expressed gene in all three strawberry infected samples was the gene encoding for the glycosyl glycosyl hydrolase GH 7, KXH38331 gene with CAZys annotations (**Table 2**; **Supplementary Figure S1**). Glycosyl hydrolase GH 7, KXH38331 targets chitins as a substrate and just like LysM proteins, these genes are differentially expressed at early timepoints. This expression pattern might be related to the protection from plant chitinases to establish biotrophy in the beginning. Another glycosyl hydrolase GH family member, KXH53384, which encodes for Glycosyl hydrolase GH 18 family is highly conserved on fungi and oomycetes, and in *Phytophthora sojae*, its knockouts were found to have severely reduced virulence in soybeans (Tan et al., 2020) (**Supplementary Table S1**).

Along with many pathogenicity related genes, SM genes such as Cytochrome P450s, oxidoreductases and transporters are also highly expanded in *Colletotrichum* species genomes (Liang et al., 2018). The expression of these genes was generally observed at the later timepoints in all three samples (**Table 2**).

Cytochrome P450s are a large group of genes associated with metabolism pathways for the degradation of xenobiotic compounds or detoxification of host metabolites (Allan et al., 2019). The expression pattern of Cytochrome P450s in the later stages might show their functioning in the detoxification of strawberry defense metabolites produced against fungi. Transmembrane transporters are also associated with the detoxification of antifungal metabolites and plant-produced toxins. Especially two types of transporters, ATP-binding Cassette (ATP) and Major Facilitator Superfamily (MFS), are well known for these functions in plants. While ABC transporters are more related to conidiation and the resistance to abiotic stress or fungicides, MFS transporters are more related to sugar uptake at the necrotrophic phase (Dubrulle et al., 2020; Kim et al., 2014). Correspondingly, ABC gene family members had more significant upregulation in the unwounded leaves compared to the fruit or wounded leaf samples, which might be explained with the secondary conidiation in the leaves (**Supplementary Table S1**) (Dowling et al., 2020). On the other hand, some MFS gene family members, such as KXH44404, had a very high expression, especially at the later timepoints in the fruits. Oxidoreductases can increase virulence through various functions like an oxidative breakdown of cell wall components, detoxifying plant produced toxins or metabolites, and regulating the pH in the host tissues (Liang et al., 2018). NADPH2:quinone reductase is an oxidoreductase enzyme that converts cytotoxic quinone compounds to less harmful quinol (Ryan et al., 2014). One particular NADPH2:quinone reductase gene, KXH57053, showed very significant upregulation at the later stages and at the 1 dpi in the unwounded leaves (**Table 2**). Besides the various functions oxidoreductases contribute to the pathogenicity, this enzyme might be related to the resistance to Quinone outside inhibitors (QoI) reported by Forcelini et al. (2016) in *Colletotrichum acutatum* s.l (Forcelini et al., 2016).

Necrotic-like proteins such as NUDIX or Necrosis-inducing Protein (NIP) are considered as the trademarks of necrotrophy as they are specifically expressed at the switch to the necrotrophic lifestyle. The expression pattern of NIP gene KXH60658 was consistent with this, and it was very highly regulated at the later time points. Although the expression started earlier in the wounded leaves (at 2 dpi) compared to the unwounded leaves (at 5 dpi) and the fruits (at 3 dpi), it can be explained with the possibility that wounding might have triggered the switch to happen earlier. NUDIX domain-containing gene KXH45695 had similar patterns in the wounded (at 3 dpi) and the unwounded leaves (at 3 dpi) but it surprisingly seemed to be expressed at all timepoints in the fruit (**Table 2**). The same phenomenon was observed by Dubrulle et al. (2020) in *Colletotrichum lupini* (another *C. acutatum* s.l. member) during its infection in lupins and it might suggest additional roles of NUDIX besides switching to necrotrophy.

Despite it has not been directly shown to contribute to virulence yet, tannase gene KXH55186 might require special attention based on being in the top upregulated genes in both leaf and fruit samples at almost all the time points (**Table 2**). Together KXH55186, and KXH64601 had a different expression pattern compared to the other tannase family members (**Figure 4**). To understand the differential expression pattern among the tannase genes we analyzed their active sites and performed phylogenetic analysis. Active site prediction by I-TASSER confirmed the presence of the characteristic catalytic triad in KXH64601 and KXH55186 (**Figure 5**). Meanwhile the multiple alignment analysis showed the differences in the conserved pentapeptide which includes the serine residue of the catalytic triad. The alignment results yielded a clade including KXH55186 and KXH64601, together with sscle_08g067140 and sscle_10g076570, which were previously found to be differentially expressed during *S. sclerotiorum* infections. This situation might be explained by the specific motifs in the conserved pentapeptides of tannase enzymes. Like the feruloyl esterases, tannase proteins commonly share a catalytic triad which consists of Ser-Asp-His (S-D-H) residues that are essential for enzymatic activity (Koseki et al., 2017; Wong, 2006). A study analyzed the tannase protein sequence from *Botryotinia fuckeliana*, a plant pathogen, and its tertiary structure prediction showed the triad with (S-H-D) residues (Gasse, 2014). To illustrate the necessity of the catalytic triad, Hansen and Marcatili (2020), replaced the serine residue of the triad with an alanine and the enzyme was completely inactivated as a result (Hansen and Marcatili, 2020). Furthermore, the serine residue of this triad is located in the center of a conserved pentapeptide motif; Gly-X-Ser-X-Gly (GXSXG) and previously analyzed tannase protein sequences showed that there are phenotypic differences between bacterial and fungal or yeast tannases in this motif (Borrego-Terrazas et al., 2014; Yao et al., 2014). Gly-Cys-Ser-Asp-Gly (GCSDG) and Gly-Cys-Ser-Glu-Gly (GCSEG) phenotypes are specifically conserved within fungi and yeast, respectively, while Gly-Cys-Ser-Thr-Gly (GCSTG) phenotype is highly conserved in bacteria (Borrego-Terrazas et al., 2014).The four protein sequences within the clade of the upregulated tannases had either “GCSDG” or “GCSEG” pentapeptide which were specific phenotypes for fungi and yeast (**Figures 6**, **7**). On the other hand, most of the tannases that were not differentially expressed during the infections had the bacterial specific phenotype with “GCSTG” pentapeptide. Therefore, presence of fungal- or yeast-specific pentapeptide in tannase enzymes might hint their special adaptation for certain niche during infections.

According to the GO enrichment analysis, the term ‘hydrolase activity’ was notably over-represented in among all upregulated genes encoding for secreted proteins, being the most enriched in the infected wounded leaves, and the second most enriched in the infected unwounded leaves and fruits (**Figure 8**). Hydrolase activity encompasses peptidase and carbohydrate-degrading activities, which have been reported to be closely associated with fungal pathogenicity (Gan et al., 2016; Human et al., 2020). Furthermore, some of the genes associated with the hydrolase activity encode CWDEs (Zhang et al., 2020). Therefore, these results align with the strong association of CWDEs and their characterized roles in pathogenicity across *Colletotrichum* species.

## Conclusion

In this study, we presented a set of characterized and novel genes that serve as valuable resources for comprehending the infectious mechanism of *Colletotrichum nymphaeae* at the molecular level. Our results suggest that *C. nymphaeae* exhibits dynamical gene expression patterns associated with various functions throughout the stages of the infection. Specifically, the early stages of the infection demonstrated significant upregulations of chitinase genes with LysM domains or ABC transporter genes, indicating potential roles in chitin sequestering, suppression of the host defense system, and in appresoria development. Meanwhile, after switching to necrotrophy, we observed a sharp increase in the expression of genes encoding for CAZymes, secondary metabolites SM-related, and necrotic-like genes. Nevertheless, our findings also revealed an unexpected early upregulation of a NUDIX gene, contradicting its established role in the lifestyle switch, thus indicating potential undiscovered roles and underscoring the need for deeper inquiry into its functional analysis. Our findings also strongly suggest that tannase enzymes may play a pivotal role in pathogenicity, not only within *Colletotrichum* spp. but across a broad spectrum of phytopathogens. Consequently, it is imperative to elucidate their roles in pathogenicity through further assays. Nonetheless, the functional characterization of tannase and other upregulated genes from *C.nymphaeae* is essential to validate their contributions to anthracnose disease mechanisms. To achieve this, techniques such as gene knockout or RNA interference can be applied to measure differences in pathogen virulence.

## Data Availability

The datasets presented in this study can be found in online repositories. The names of the repository/repositories and accession number(s) can be found below: https://www.ncbi.nlm.nih.gov/, PRJNA1074080.

## Supplementary material

Supplementary Table 2 Primers used for qPCR validation of *Colletotrichum nymphaeae in planta* expressed genes.

Supplementary Table 3 Summary of Gene Ontology GO Terms significantly enriched in strawberry tissues infected by *Colletotrichum nymphaeae* during infection in the wounded leaves, unwounded leaves and fruit.

Supplementary Table 4 Summary of differentially expressed genes (DEGs) found in *Fragaria* x *ananassa* wounded leaf, unwounded leaf and fruit samples infected with *C. nymphaeae*.

Supplementary Figure 1 Heatmap showing *Colletotrichum nymphaeae* candidate genes expressed in strawberry tissues and encoding for secreted proteins with reported roles in pathogenicity in *Colletotrichum* species and other fungal plant pathogens.

Predicted active side residues of two *Colletotrichum nymphaeae* Tannase proteins, by I-TASSER. **(A)** Predicted active side residues of CNYM01_13686 (KXH64601). **(B)** Predicted active site residues of CNYM01_08295 (KXH55186).

Supplementary Figure 3 Comparison of RNAseq gene expression and reverse transcription-quantitative polymerase chain reaction (RT-qPCR) validation of six candidate genes of *Colletotrichum nymphaeae*. **(A)** gene CNYM01_02731 (KXH43592) encoding for multicopper oxidase protein. **(B)** gene CNYM01_07521 (KXH38331) encoding for glycosyl hydrolase family 7 protein. **(C)** gene CNYM01_06500 (KXH30597) encoding for endochitinase protein. **(D)** gene CNYM01_11346 (KXH38857) encoding for cytochrome P450 protein. **(E)** gene CNYM01_03788 (KXH42765) encoding for pectate lyase protein. **(F)** gene CNYM01_07125 (KXH45695) encoding for NUDIX protein. The light green, light yellow and light pink colored bars are used to illustrate RNAseq log2 fold change values in wounded leaves, unwounded leaves, and fruits, respectively. The dark green, dark brown and dark red colored bars are used to illustrate the RT-qPCR fold change mean values in wounded leaves, unwounded leaves, and fruits, respectively.

## References

[B1] Aguilar C. N. Rodríguez R. Gutiérrez-Sánchez G. Augur C. Favela-Torres E. Prado-Barragan L. A.. (2007). Microbial tannases: advances and perspectives. Appl. Microbiol. Biotechnol. 76, 47–59. doi: 10.1007/s00253-007-1000-2 17530245

[B2] Allan J. Regmi R. Denton-Giles M. Kamphuis L. G. Derbyshire M. C. (2019). The host generalist phytopathogenic fungus Sclerotinia sclerotiorum differentially expresses multiple metabolic enzymes on two different plant hosts. Sci. Rep. 9, 7–11. doi: 10.1038/s41598-019-56396-w PMC6934579 31882688

[B3] Almagro Armenteros J. J. Tsirigos K. D. Sonderby C. K. Petersen T. N. Winther O. Brunak S.. (2019). SignalP 5.0 improves signal peptide predictions using deep neural networks. Nat. Biotechnol. 37, 420–423. doi: 10.1038/s41587-019-0036-z 30778233

[B4] Amil-Ruiz F. Blanco-Portales R. Muñoz-Blanco J. Caballero J. L. (2011). The strawberry plant defense mechanism: A molecular review. Plant Cell Physiol. 52, 1873–1903. doi: 10.1093/pcp/pcr136 21984602

[B5] Baraldi E. Coller E. Zoli L. Cestaro A. Tosatto S. C. Zambelli B. (2015). Unfoldome variation upon plant-pathogen interactions: strawberry infection by *Colletotrichum acutatum* . Plant Mol. Biol. 89, 49–65. doi: 10.1007/s11103-015-0353-7 26245354

[B6] Baroncelli R. Amby D. B. Zapparata A. Sarrocco S. Vannacci G. Le Floch G.. (2016). Gene family expansions and contractions are associated with host range in plant pathogens of the genus *Colletotrichum* . BMC Genomics 17, 1–2. doi: 10.1186/s12864-016-2917-6 PMC4974774 27496087

[B7] Baroncelli R. Talhinhas P. Pensec F. Sukno S. A. Le Floch G. Thon M. R. (2017). The *Colletotrichum acutatum* species complex as a model system to study evolution and host specialization in plant pathogens. Front. Microbiol. 8. doi: 10.3389/fmicb.2017.02001 PMC5641571 29075253

[B8] Baroncelli R. Zapparata A. Sarrocco S. Sukno S. A. Lane C. R. Thon M. R.. (2015). Molecular diversity of anthracnose pathogen populations associated with UK strawberry production suggests multiple introductions of three different *Colletotrichum* species. PloS One 10, e0129140. doi: 10.1371/journal.pone.0129140 26086351 PMC4472692

[B9] Blackman L. M. Cullerne D. P. Hardham A. R. (2014). Bioinformatic characterisation of genes encoding cell wall degrading enzymes in the Phytophthora parasitica genome. BMC Genomics 15, 785. doi: 10.1186/1471-2164-15-785 25214042 PMC4176579

[B10] Borrego-Terrazas J. A. Lara-Victoriano F. Flores-Gallegos A. C. Veana F. Aguilar C. N. Rodríguez-Herrera R. (2014). Nucleotide and amino acid variations of tannase gene from different Aspergillus strains. Can. J. Microbiol. 60, 509–516. doi: 10.1139/cjm-2014-0163 25065666

[B11] Cheah L. H. Soteros J. J. (1984). Control of black fruit rot of strawberry, NZ Weed Pest Control Soc., Inc., Hastings. (New Zealand: New Zealand Plant Protection) 160–162. doi: 10.30843/nzpp.1984.37

[B12] Crouch J. O’Connell R. Gan P. Buiate E. Torres M. F. Beirn L.. (2014). The Genomics of Colletotrichum (Springer, Berlin, Heidelberg), 69–102. doi: 10.1007/978-3-662-44053-7_3

[B13] Debode J. Van Hemelrijck W. Xu X. M. Maes M. Creemers P. Heungens K. (2015). Latent entry and spread of Colletotrichum acutatum (species complex) in strawberry fields. Plant Pathol. 64, 385–395. doi: 10.1111/ppa.12247

[B14] De Silva D. D. Crous P. W. Ades P. K. Hyde K. D. Taylor P. W. J. (2017). Life styles of Colletotrichum species and implications for plant biosecurity. Fungal Biol. Rev. 31, 155–168. doi: 10.1016/j.fbr.2017.05.001

[B15] Dowling M. Peres N. Villani S. Schnabel G. (2020). Managing colletotrichum on fruit crops: A “Complex” Challenge. Plant Dis. 104, 2301–2316. doi: 10.1094/pdis-11-19-2378-fe 32689886

[B16] Dubrulle G. Picot A. Madec S. Corre E. Pawtowski A. Baroncelli R.. (2020). Deciphering the Infectious Process of Colletotrichum lupini in Lupin through Transcriptomic and Proteomic Analysis. Microorganisms 8, 1621. doi: 10.3390/microorganisms8101621 33096724 PMC7589765

[B17] Forcelini B. B. Gonçalves F. P. Peres N. A. (2017). Effect of inoculum concentration and interrupted wetness duration on the development of anthracnose fruit rot of strawberry. Plant Dis. 101, 372–377. doi: 10.1094/pdis-08-16-1175-re 30681921

[B18] Forcelini B. B. Rebello C. S. Wang N. Y. Peres N. A. (2018). Fitness, competitive ability, and mutation stability of isolates of *Colletotrichum acutatum* from strawberry resistant to QoI fungicides. Phytopathology 108, 462–468. doi: 10.1094/PHYTO-09-17-0296-R 29135359

[B19] Forcelini B. B. Seijo T. E. Amiri A. Peres N. A. (2016). Resistance in strawberry isolates of *Colletotrichum acutatum* from Florida to quinone-outside inhibitor fungicides. Plant Dis. 100, 2050–2056. doi: 10.1094/PDIS-01-16-0118-RE 30683005

[B20] Freeman S. (2008). Management, survival strategies, and host range of colletotrichum acutatum on strawberry. HortScience 43, 66–68. doi: 10.21273/hortsci.43.1.66

[B21] Gan P. Ikeda K. Irieda H. Narusaka M. O’Connell R. J. Narusaka Y.. (2013). Comparative genomic and transcriptomic analyses reveal the hemibiotrophic stage shift of Colletotrichum fungi. New Phytol. 197, 1236–1249. doi: 10.1111/nph.12085 23252678

[B22] Gan P. Narusaka M. Kumakura N. Tsushima A. Takano Y. Narusaka Y.. (2016). Genus-wide comparative genome analyses of colletotrichum species reveal specific gene family losses and gains during adaptation to specific infection lifestyles. Genome Biol. Evol. 8, 1467–1481. doi: 10.1093/gbe/evw089 27189990 PMC4898803

[B23] Gasse M. (2014). The tannase gene: metaphylogenomics, global distribution and presence in the midgut flora of the forest tent caterpillar malacosoma disstria hübner. Available online at: https://scholar.google.com/scholar?cites=8104480370044281947&as_sdt=40005&sciodt=0,10&hl=enhttps://core.ac.uk/download/pdf/211517128.pdf.

[B24] Guidarelli M. Carbone F. Mourgues F. Perrotta G. Rosati C. Bertolini P.. (2011). Colletotrichum acutatum interactions with unripe and ripe strawberry fruit and differential responses at histological and transcriptional levels. Plant Pathol. 60, 685–697. doi: 10.1111/j.1365-3059.2010.02423.x

[B25] Hannon G. J. (2010). FASTX-toolkit. Available online at: https://scholar.google.com/scholar?hl=en&as_sdt=0,10&cluster=11504550324592496137.

[B26] Hansen E. B. Marcatili P. (2020). Modeled structure of the cell envelope proteinase of lactococcus lactis. Front. Bioengineering Biotechnol. 8. doi: 10.3389/fbioe.2020.613986 PMC7783315 33415101

[B27] Higuera J. J. Garrido-Gala J. Lekhbou A. Arjona-Girona I. Amil-Ruiz F. Mercado J. A.. (2019). The strawberry faWRKY1 transcription factor negatively regulates resistance to colletotrichum acutatum in fruit upon infection. Front. Plant Sci. 10. doi: 10.3389/fpls.2019.00480 PMC6482226 31057583

[B28] Human M. P. Berger D. K. Crampton B. G. (2020). Time-course RNAseq reveals exserohilum turcicum effectors and pathogenicity determinants. Front. Microbiol. 11. doi: 10.3389/fmicb.2020.00360 PMC7099616 32265851

[B29] Kim K.-T. Jeon J. Choi J. Cheong K. Song H. Choi G.. (2016). Kingdom-wide analysis of fungal small secreted proteins (SSPs) reveals their potential role in host association. Front. Plant Sci. 7. doi: 10.3389/fpls.2016.00186 PMC4759460 26925088

[B30] Kim S. Park S.-Y. Kim H. Kim D. Lee S.-W. Kim H. T.. (2014). Isolation and characterization of the colletotrichum acutatum ABC transporter caABC1. Plant Pathol. J. 30, 375–383. doi: 10.5423/ppj.oa.08.2014.0077 25506302 PMC4262290

[B31] Kim D. Pertea G. Trapnell C. Pimentel H. Kelley R. Salzberg S. L. (2013). TopHat2: accurate alignment of transcriptomes in the presence of insertions, deletions and gene fusions. Genome Biol. 14, R36. doi: 10.1186/gb-2013-14-4-r36 23618408 PMC4053844

[B32] Kleemann J. Rincon-Rivera L. J. Takahara H. Neumann U. Ver Loren van Themaat E. van der Does H. C.. (2012). Sequential delivery of host-induced virulence effectors by appressoria and intracellular hyphae of the phytopathogen *Colletotrichum higginsianum* . PloS Pathog. 8, e1002643. doi: 10.1371/journal.ppat.1002643 22496661 PMC3320591

[B33] Koseki T. Otsuka M. Mizuno T. Shiono Y. (2017). Mutational analysis of Kex2 recognition sites and a disulfide bond in tannase from Aspergillus oryzae. Biochem. Biophys. Res. Commun. 482, 1165–1169. doi: 10.1016/j.bbrc.2016.12.006 27919681

[B34] Langmead B. Salzberg S. L. (2012). Fast gapped-read alignment with Bowtie 2. Nat. Methods 9, 357–359. doi: 10.1038/nmeth.1923 22388286 PMC3322381

[B35] Liang X. Wang B. Dong Q. Li L. Rollins J. A. Zhang R.. (2018). Pathogenic adaptations of Colletotrichum fungi revealed by genome wide gene family evolutionary analyses. PloS One 13, e0196303. doi: 10.1371/journal.pone.0196303 29689067 PMC5915685

[B36] Livak K. J. Schmittgen T. D. (2001). Analysis of relative gene expression data using real-time quantitative PCR and the 2(-Delta Delta C(T)) Method. Methods 25, 402–408. doi: 10.1006/meth.2001.1262 11846609

[B37] Love M. I. Huber W. Anders S. (2014). Moderated estimation of fold change and dispersion for RNA-seq data with DESeq2. Genome Biol. 15, 1. doi: 10.1186/s13059-014-0550-8 PMC4302049 25516281

[B38] Mamaní A. Filippone M. P. Grellet C. Welin B. Castagnaro A. P. Ricci J. C. D. (2012). Pathogen-induced accumulation of an ellagitannin elicits plant defense response. Molecular Plant-Microbe Interactions. 25, 1430–1439. doi: 10.1094/mpmi-12-11-0306 22934564

[B39] Mertely J. C. Legard D. E. (2004). Detection, isolation, and pathogenicity of colletotrichum spp. from strawberry petioles. Plant Dis. 88, 407–412. doi: 10.1094/pdis.2004.88.4.407 30812623

[B40] Nielsen H. Engelbrecht J. Brunak S. von Heijne G. (1997). Identification of prokaryotic and eukaryotic signal peptides and prediction of their cleavage sites. Protein Eng. 10, 1–6. doi: 10.1093/protein/10.1.1 9051728

[B41] O’Connell R. J. Thon M. R. Hacquard S. Amyotte S. G. Kleemann J. Torres M. F.. (2012). Lifestyle transitions in plant pathogenic *Colletotrichum* fungi deciphered by genome and transcriptome analyses. Nat. Genet. 44, 1060–1065. doi: 10.1038/ng.2372 22885923 PMC9754331

[B42] Olson S. A. (1994). MacVector: An Integrated Sequence Analysis Program for the Macintosh. In: Computer Analysis of Sequence Data. Methods in Molecular Biology (Springer, Totowa, NJ: Humana Press) 25, 195–201. doi: 10.1385/0-89603-276-0:195 8004165

[B43] Pierleoni A. Martelli P. L. Casadio R. (2008). PredGPI: a GPI-anchor predictor. BMC Bioinf. 9, 392. doi: 10.1186/1471-2105-9-392 PMC2571997 18811934

[B44] Prusky D. Lichter A. (2007). Activation of quiescent infections by postharvest pathogens during transition from the biotrophic to the necrotrophic stage. FEMS Microbiol. Lett. 268, 1–8. doi: 10.1111/j.1574-6968.2006.00603.x 17227463

[B45] Putri G. H. Anders S. Pyl P. T. Pimanda J. E. Zanini F. (2022). Analysing high-throughput sequencing data in Python with HTSeq 2.0. Bioinformatics 38, 2943–2945. doi: 10.1093/bioinformatics/btac166 35561197 PMC9113351

[B46] Reges de Sena A. Claúdia de Barros Dos Santos A. Gouveia M. J. Figueira de Mello M. R. Leite T. C. C. Moreira K. A.. (2014). Production, characterization and application of a thermostable tannase from pestalotiopsis guepinii URM 7114. Food Technol. Biotechnol. 52, 459–467. doi: 10.17113/ftb.52.04.14.3743 27904319 PMC5079146

[B47] Ridzuan R. Rafii M. Ismail S. Mohammad Yusoff M. Miah G. Usman M. (2018). Breeding for anthracnose disease resistance in chili: progress and prospects. Int. J. Mol. Sci. 19, 3122. doi: 10.3390/ijms19103122 30314374 PMC6213496

[B48] Ristinmaa A. S. Coleman T. Cesar L. Langborg Weinmann A. Mazurkewich S. Brändén G.. (2022). Structural diversity and substrate preferences of three tannase enzymes encoded by the anaerobic bacterium Clostridium butyricum. J. Biol. Chem. 298, 101758. doi: 10.1016/j.jbc.2022.101758 35202648 PMC8958541

[B49] Ryan A. Kaplan E. Nebel J.-C. Polycarpou E. Crescente V. Lowe E.. (2014). Identification of NAD(P)H quinone oxidoreductase activity in azoreductases from P. aeruginosa: azoreductases and NAD(P)H quinone oxidoreductases belong to the same FMN-dependent superfamily of enzymes. PloS One 9, e98551. doi: 10.1371/journal.pone.0098551 24915188 PMC4051601

[B50] Shang S. Wang B. Zhang S. Liu G. Liang X. Zhang R.. (2020). A novel effector CfEC92 of *Colletotrichum fructicola* contributes to glomerella leaf spot virulence by suppressing plant defences at the early infection phase. Mol. Plant Pathol. 21, 936–950. doi: 10.1111/mpp.12940 32512647 PMC7279981

[B51] Sharma K. P. (2019). Tannin degradation by phytopathogen’s tannase: A Plant’s defense perspective. Biocatalysis Agric. Biotechnol. 21, 101342. doi: 10.1016/j.bcab.2019.101342

[B52] Sperschneider J. Catanzariti A. M. DeBoer K. Petre B. Gardiner D. M. Singh K. B.. (2017). LOCALIZER: subcellular localization prediction of both plant and effector proteins in the plant cell. Sci. Rep. 7, 44598. doi: 10.1038/srep44598 28300209 PMC5353544

[B53] Sperschneider J. Dodds P. N. Gardiner D. M. Singh K. B. Taylor J. M. (2018). Improved prediction of fungal effector proteins from secretomes with EffectorP 2.0. Mol. Plant Pathol. 19, 2094–2110. doi: 10.1111/mpp.12682 29569316 PMC6638006

[B54] Sreenivasaprasad S. Talhinhas P. (2005). Genotypic and phenotypic diversity in *Colletotrichum acutatum*, a cosmopolitan pathogen causing anthracnose on a wide range of hosts. Mol. Plant Pathol. 6, 361–378. doi: 10.1111/j.1364-3703.2005.00291.x 20565664

[B55] Staňková B. Víchová J. Pokorný R. (2011). Virulence of *Colletotrichum acutatum* isolates to several host plants. Acta Universitatis Agriculturae Silviculturae Mendelianae Brunensis 59, 161–170. doi: 10.11118/actaun201159030161

[B56] Sun J. Cao L. Li H. Wang G. Wang S. Li F.. (2019). Early responses given distinct tactics to infection of Peronophythora litchii in susceptible and resistant litchi cultivar. Sci. Rep. 9, 3. doi: 10.1038/s41598-019-39100-w 30808947 PMC6391439

[B57] Sützl L. Laurent C. V. F. P. Abrera A. T. Schütz G. Ludwig R. Haltrich D. (2018). Multiplicity of enzymatic functions in the CAZy AA3 family. Appl. Microbiol. Biotechnol. 102, 2477–2492. doi: 10.1007/s00253-018-8784-0 29411063 PMC5847212

[B58] Takahara H. Hacquard S. Kombrink A. Hughes H. B. Halder V. Robin G. P.. (2016). *Colletotrichum higginsianum* extracellular LysM proteins play dual roles in appressorial function and suppression of chitin-triggered plant immunity. New Phytol. 211, 1323–1337. doi: 10.1111/nph.13994 27174033

[B59] Tan X. Hu Y. Jia Y. Hou X. Xu Q. Han C.. (2020). A conserved glycoside hydrolase family 7 cellobiohydrolase psGH7a of phytophthora sojae is required for full virulence on soybean. Front. Microbiol. 11. doi: 10.3389/fmicb.2020.01285 PMC7343703 32714289

[B60] Tsushima A. Narusaka M. Gan P. Kumakura N. Hiroyama R. Kato N.. (2021). The conserved colletotrichum spp. Effector candidate CEC3 induces nuclear expansion and cell death in plants. Front. Microbiol. 12:682155. doi: 10.3389/fmicb.2021.682155 34539598 PMC8446390

[B61] Wang N.-Y. Forcelini B. B. Peres N. A. (2019). Anthracnose fruit and root necrosis of strawberry are caused by a dominant species within the *Colletotrichum acutatum* species complex in the United States. Phytopathology 109, 1293–1301. doi: 10.1094/phyto-12-18-0454-r 30852972

[B62] Wang Z. Gerstein M. Snyder M. (2009). RNA-Seq: a revolutionary tool for transcriptomics. Nat. Rev. Genet. 10, 57–63. doi: 10.1038/nrg2484 19015660 PMC2949280

[B63] Wilkinson L. (2011). ggplot2: elegant graphics for data analysis by WICKHAM, H. Biometrics 67, 678–679. doi: 10.1111/j.1541-0420.2011.01616.x

[B64] Wong D. W. (2006). Feruloyl esterase: a key enzyme in biomass degradation. Appl. Biochem. Biotechnol. 133, 87–112. doi: 10.1385/abab:133:2:87 16702605

[B65] Yao J. Guo G. S. Ren G. H. Liu Y. H. (2014). Production, characterization and applications of tannase. J. Mol. Catalysis B: Enzymatic 101, 137–147. doi: 10.1016/j.molcatb.2013.11.018

[B66] Zeilinger S. Gupta V. K. Dahms T. E. S. Silva R. N. Singh H. B. Upadhyay R. S.. (2016). Friends or foes? Emerging insights from fungal interactions with plants. FEMS Microbiol. Rev. 40, 182–207. doi: 10.1093/femsre/fuv045 26591004 PMC4778271

[B67] Zhang X. Cheng W. Feng Z. Zhu Q. Sun Y. Li Y.. (2020). Transcriptomic analysis of gene expression of Verticillium dahliae upon treatment of the cotton root exudates. BMC Genomics 21, 7–8. doi: 10.1186/s12864-020-6448-9 PMC7017574 32050898

[B68] Zhang L. Huang X. He C. Zhang Q. Y. Zou X. Duan K.. (2018b). Novel fungal pathogenicity and leaf defense strategies are revealed by simultaneous transcriptome analysis of *Colletotrichum fructicola* and strawberry infected by this fungus. Front. Plant Sci. 9. doi: 10.3389/fpls.2018.00434 PMC5996897 29922301

[B69] Zhang H. Yohe T. Huang L. Entwistle S. Wu P. Yang Z.. (2018a). dbCAN2: a meta server for automated carbohydrate-active enzyme annotation. Nucleic Acids Res. 46, W95–w101. doi: 10.1093/nar/gky418 29771380 PMC6031026

[B70] Zheng W. Zhang C. Li Y. Pearce R. Bell E. W. Zhang Y. (2021). Folding non-homologous proteins by coupling deep-learning contact maps with I-TASSER assembly simulations. Cell Rep. Methods 1, 1. doi: 10.1016/j.crmeth.2021.100014 PMC8336924 34355210

