# Supplementary figures and images for: Transcriptome analysis of Colletotrichum nymphaeae-Strawberry interaction reveals in planta expressed genes associated with virulence
Source: Front Plant Sci. 2025 Jan 20;15:1390926. doi: 10.3389/fpls.2024.1390926 (PMC11803528; doi:10.3389/fpls.2024.1390926)

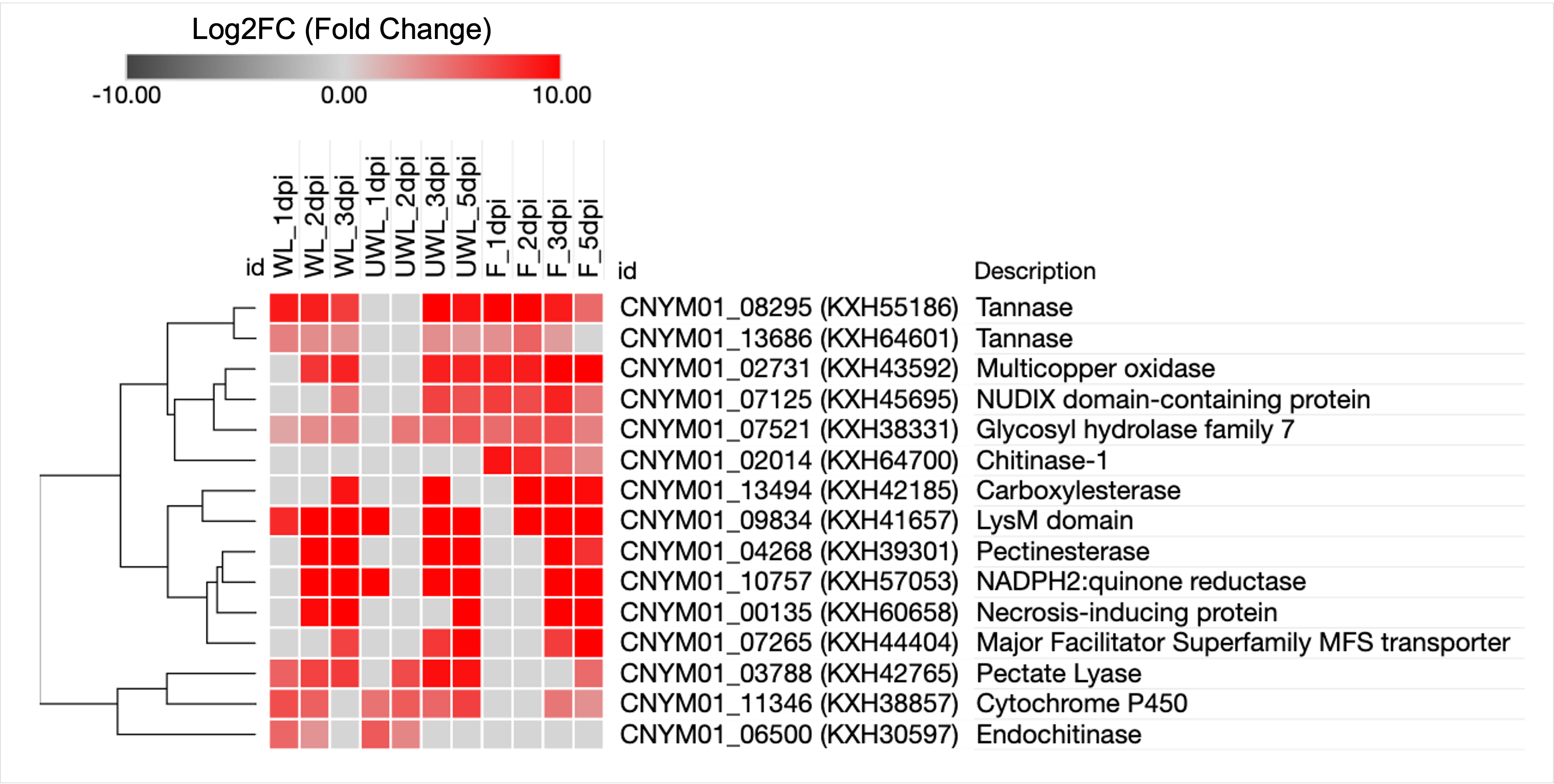

Supplement: Supplementary Figure 1 — Heatmap showing Colletotrichum nymphaeae candidate genes expressed in strawberry tissues and encoding for secreted proteins with reported roles in pathogenicity in Colletotrichum species and other fungal plant pathogens. [file Image1.tiff]

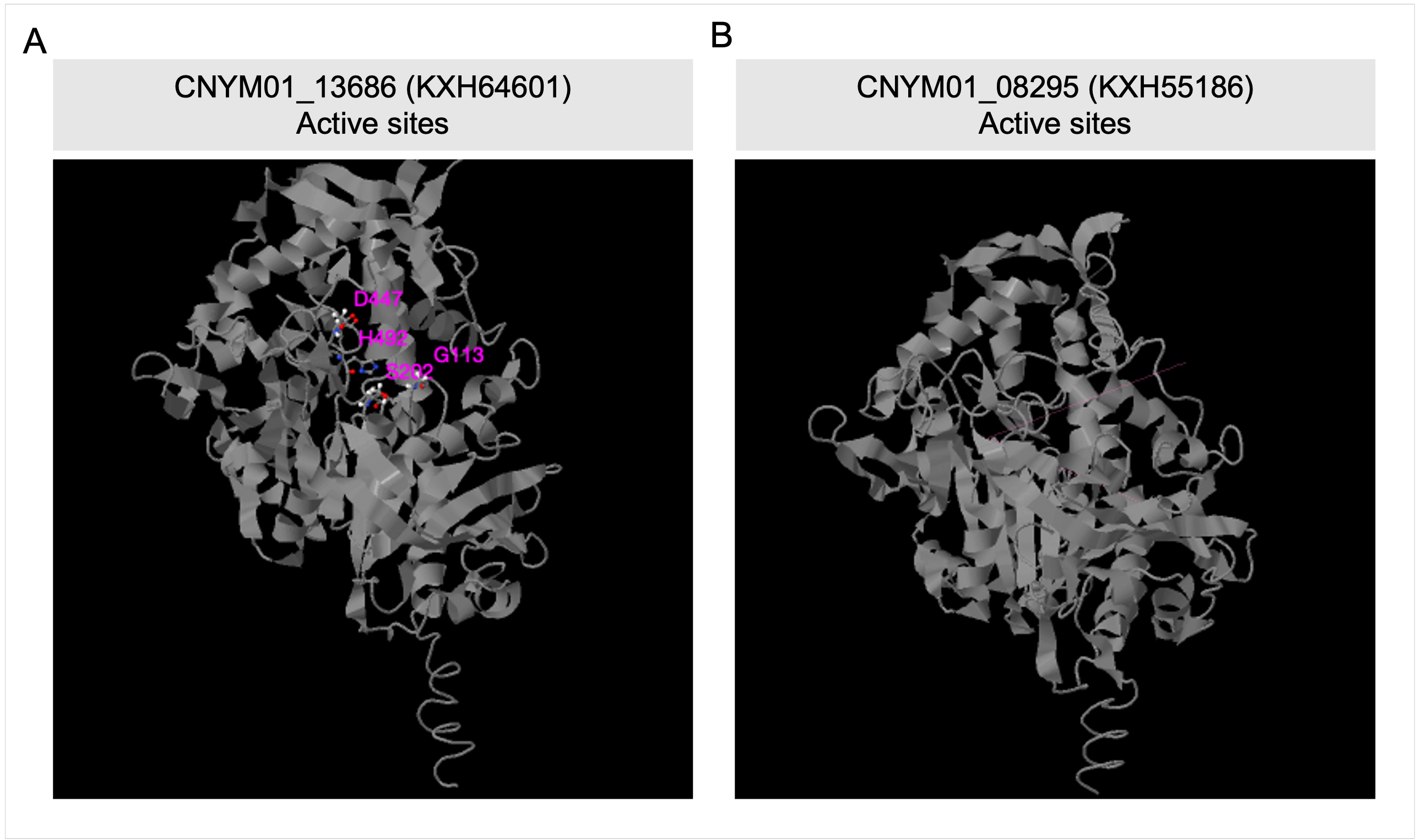

Supplement: Supplementary file 6 — Predicted active side residues of two Colletotrichum nymphaeae Tannase proteins, by I-TASSER. (A) Predicted active side residues of CNYM01_13686 (KXH64601). (B) Predicted active site residues of CNYM01_08295 (KXH55186). [file Image2.tiff]

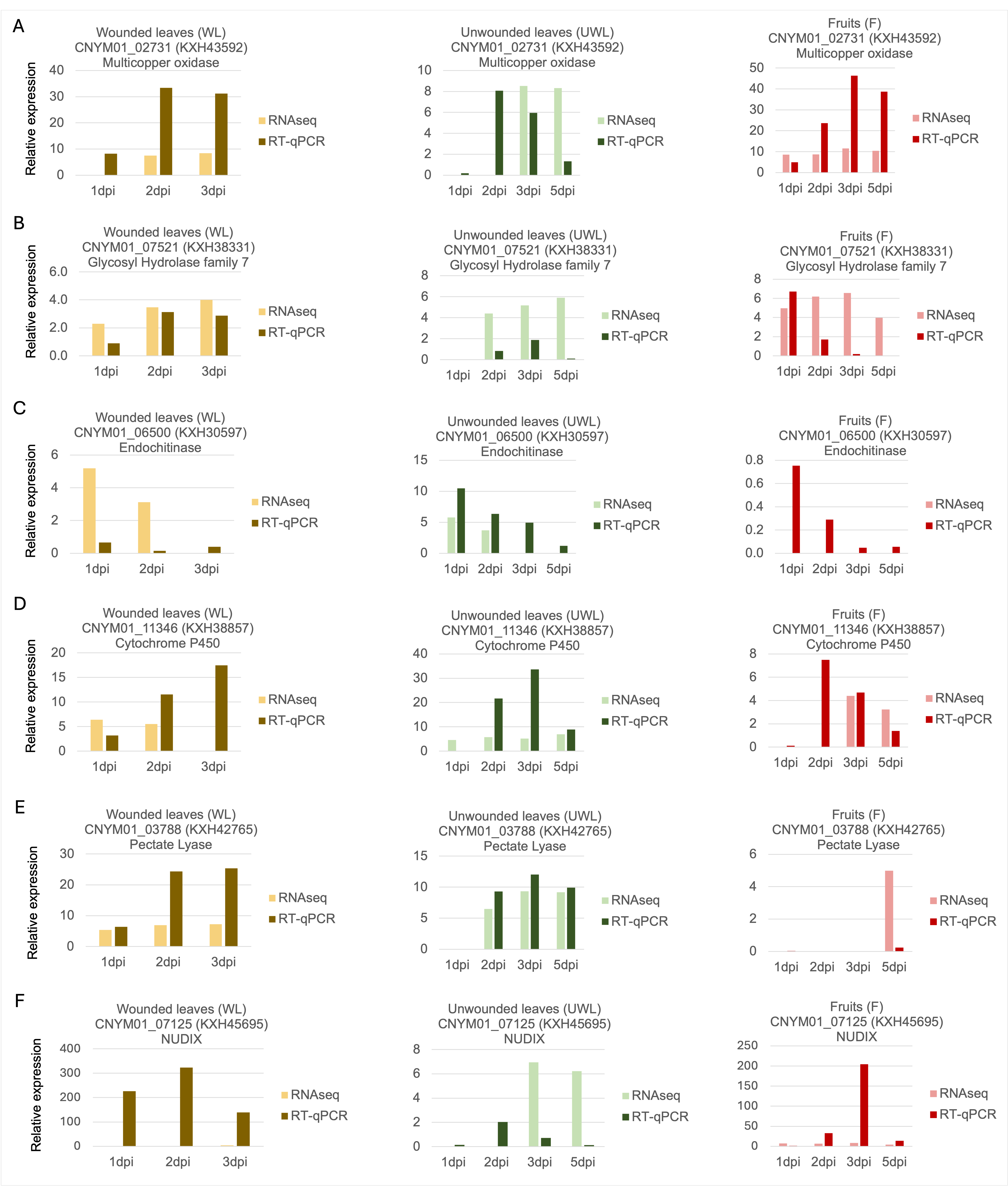

Supplement: Supplementary Figure 3 — Comparison of RNAseq gene expression and reverse transcription-quantitative polymerase chain reaction (RT-qPCR) validation of six candidate genes of Colletotrichum nymphaeae. (A) gene CNYM01_02731 (KXH43592) encoding for multicopper oxidase protein. (B) gene CNYM01_07521 (KXH38331) encoding for glycosyl hydrolase family 7 protein. (C) gene CNYM01_06500 (KXH30597) encoding for endochitinase protein. (D) gene CNYM01_11346 (KXH38857) encoding for cytochrome P450 protein. (E) gene CNYM01_03788 (KXH42765) encoding for pectate lyase protein. (F) gene CNYM01_07125 (KXH45695) encoding for NUDIX protein. The light green, light yellow and light pink colored bars are used to illustrate RNAseq log2 fold change values in wounded leaves, unwounded leaves, and fruits, respectively. The dark green, dark brown and dark red colored bars are used to illustrate the RT-qPCR fold change mean values in wounded leaves, unwounded leaves, and fruits, respectively. [file Image3.tiff]
